# Pharmacological treatment of sleep disorder phenotypes in Parkinson’s disease: a systematic review and meta-analysis of randomised controlled trials

**DOI:** 10.1007/s00415-026-14158-5

**Published:** 2026-09-24

**Authors:** Jaquelini Betta Canever, Laura Cechinel da Silva, Fernanda Samara Apio, Helena Iturvides Cimarosti

**Affiliations:** 1 Professional Master in Pharmacology, Department of Pharmacology, Federal University of Santa Catarina (UFSC), Campus Trindade, Florianópolis, Santa Catarina 88040-900 Brazil; 2 Postgraduate Program in Pharmacology, Department of Pharmacology, Federal University of Santa Catarina (UFSC), Florianópolis, Santa Catarina Brazil; 3 Postgraduate Program in Neuroscience, Federal University of Santa Catarina (UFSC), Florianópolis, Santa Catarina Brazil

**Keywords:** Meta-analysis, Parkinson’s disease, Pharmacological treatment, Randomised controlled trial, Sleep disorders

## Abstract

**Background:**

Sleep disturbances are amongst the most prevalent and disabling non-motor symptoms in Parkinson’s disease (PD), encompassing excessive daytime sleepiness (EDS), poor sleep quality, insomnia, and REM sleep behaviour disorder (RBD). The pharmacological evidence base remains fragmented across phenotypes, drug classes, and outcome instruments.

**Objectives:**

To evaluate the efficacy and safety of pharmacological interventions for sleep disorders in PD, stratified by sleep phenotype, through a systematic review and meta-analysis of randomised controlled trials (RCTs).

**Methods:**

Six databases were searched without restrictions. Eligible studies were RCTs in adults with PD and any sleep disturbance. Pooling was performed using random-effects models. Risk of bias was assessed with the Cochrane RoB 2 tool. The protocol was registered in PROSPERO (CRD420261412614).

**Results:**

Twenty-nine RCTs (1913 participants; 2002 to 2025) were included. Only 17.2% of trials were at low overall risk of bias. For EDS (k = 6), the pooled ESS estimate was directionally favourable but non-significant (MD 1.75; 95% CI −0.26 to 3.75; I^2^ = 91%), driven predominantly by a single influential crossover trial. For overall sleep quality (k = 6), pharmacological treatment produced a statistically significant improvement in PSQI scores (MD 2.66; 95% CI 1.63 to 3.69; I^2^ = 71%). For insomnia symptoms (k = 3) and RBD (k = 2), pooled estimates were non-significant with high heterogeneity (I^2^ = 96% and 99%, respectively), precluding reliable conclusions. Given this level of heterogeneity (I^2^ > 90%), pooled estimates for these two domains, as well as for EDS, are presented as exploratory only and narrative synthesis is emphasised over meta-analytic pooling. No unexpected safety signals were identified, although safety reporting was heterogeneous and quantitative pooling was not performed.

**Conclusions:**

Pharmacological treatment, particularly with chronobiotic agents and dopaminergic receptor agonists, may produce improvement in overall sleep quality (low-certainty evidence). Evidence for EDS, insomnia symptoms, and RBD remains insufficient for definitive recommendations, and pooled estimates in these domains should not be over-interpreted given their very high statistical heterogeneity. Phenotype-specific RCTs with harmonised outcome measures are needed.

**Graphical abstract:**

**Figure:**
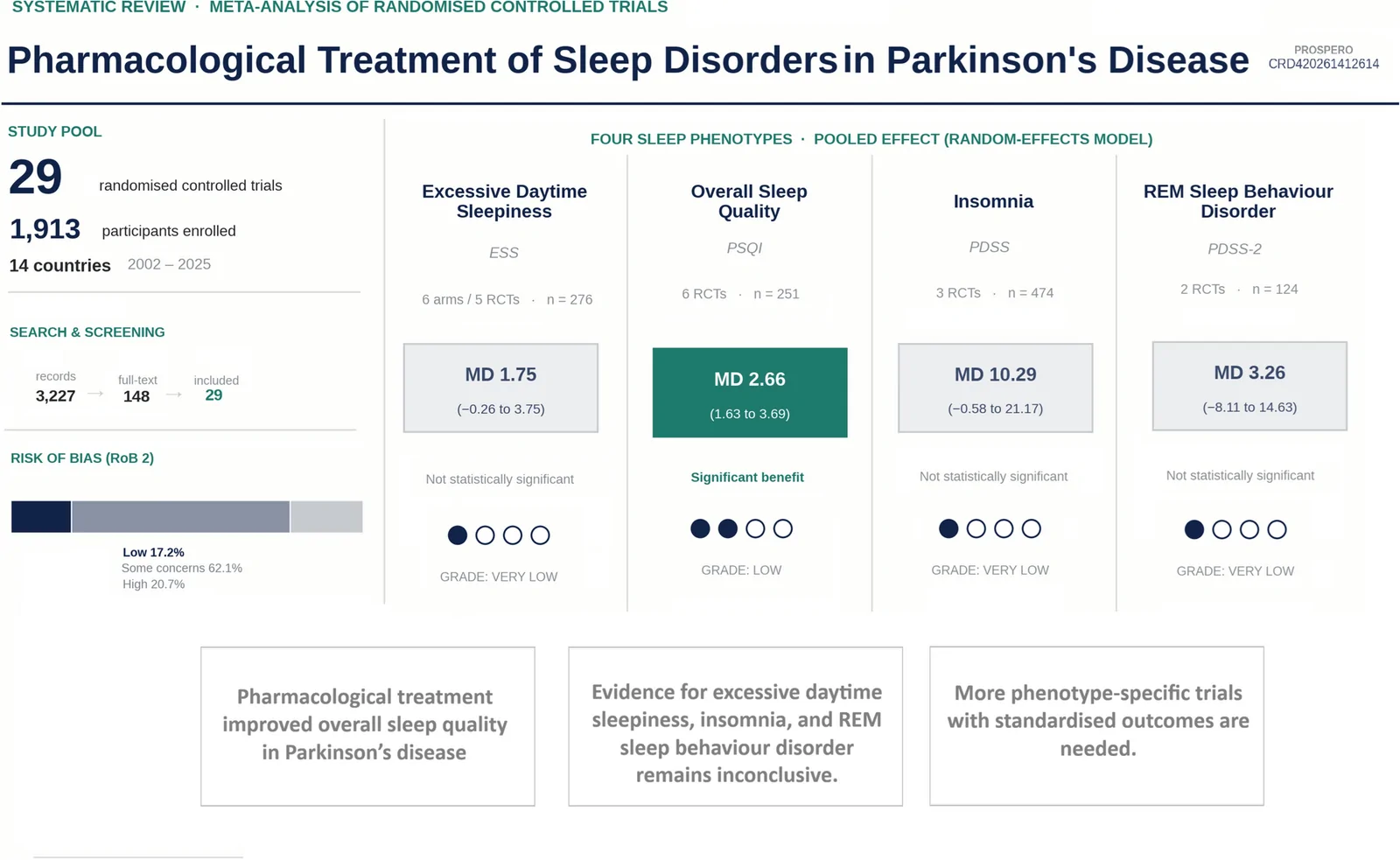

**Supplementary Information:**

The online version contains supplementary material available at https://doi.org/10.1007/s00415-026-14158-5.

## Key points

- Pharmacological treatments for sleep disturbances in Parkinson’s disease show their clearest benefit for improving overall sleep quality.
- Evidence for excessive daytime sleepiness, insomnia symptoms, and REM sleep behaviour disorder remains limited and inconsistent across randomised trials.
- Future studies should test treatments according to specific sleep phenotypes, using standardised outcome measures and better safety reporting.

**Supplementary Information:**

The online version contains supplementary material available at https://doi.org/10.1007/s00415-026-14158-5.

## Introduction

Parkinson’s disease (PD) is the second most common neurodegenerative disorder worldwide and the fastest-growing neurological condition in terms of prevalence, disability, and mortality [1]. Driven by global population ageing, the number of individuals living with PD has more than doubled over the past two decades and is projected to exceed 12 million by 2040 [2, 3]. The disease imposes a substantial burden not only on patients but also on caregivers and health systems, with costs driven largely by functional decline, hospitalization, and the management of motor and non-motor complications [3]. 

Beyond its hallmark motor features, PD encompasses a broad spectrum of non-motor symptoms, including cognitive impairment, depression, anxiety, autonomic dysfunction, pain, and olfactory loss, amongst which sleep disturbances are consistently amongst the most prevalent and disabling [2]. Sleep-related symptoms in PD are clinically heterogeneous and may include insomnia [4], excessive daytime sleepiness (EDS) [5], rapid eye movement (REM) sleep behaviour disorder (RBD) [6], restless legs syndrome [7], poor sleep quality [8], and other disturbances of the sleep–wake cycle [9]. 

The pathophysiology of sleep disturbances in PD is complex and multifactorial [1]. Degeneration of dopaminergic and non-dopaminergic pathways, α-synuclein deposition in brainstem and hypothalamic nuclei, disruption of circadian regulation, nocturnal motor symptoms, psychiatric comorbidities, autonomic dysfunction, and adverse effects of antiparkinsonian medications may all contribute to sleep impairment [10, 11]. This biological heterogeneity is clinically relevant, as different sleep phenotypes may respond differently to pharmacological interventions. For example, wake-promoting agents may be more suitable for excessive daytime sleepiness, whereas melatonergic agents, sedative-hypnotics, dopaminergic drugs, cholinesterase inhibitors, or other drug classes may target distinct mechanisms underlying insomnia, RBD, or poor sleep quality [12, 13]. 

Despite the clinical importance of sleep disturbances in PD, the pharmacological management of these symptoms remains challenging [14]. Across the three major sleep phenotypes investigated in this field, EDS, RBD, and insomnia symptoms/poor sleep quality, randomised controlled trials (RCTs) have evaluated a heterogeneous range of pharmacological interventions, spanning wake-promoting agents, dopaminergic compounds, melatonergic agents, hypnotics, antidepressants, cannabinoids, and others, as systematically mapped in the present review. However, the evidence remains fragmented across sleep phenotypes, drug classes, outcome measures, intervention durations, and study designs. Previous systematic reviews have either focused on a single pharmacological agent or used conventional pairwise meta-analytic approaches that may not fully account for dependency amongst multiple outcomes, multiple treatment arms, repeated time points, or crossover designs within the same trial [15–17]. 

Determining the most effective pharmacological strategy for sleep disturbances in PD remains challenging, as the available evidence is dispersed across randomised trials that differ in sleep phenotypes, pharmacological classes, outcome measures, treatment durations, and study designs. Conventional interpretation of individual trials is therefore limited by clinical and methodological heterogeneity, as well as by the small number of studies available for several sleep-related outcomes. Accordingly, we conducted this systematic review and meta-analysis of RCTs to evaluate the efficacy and safety of pharmacological treatments for sleep disturbances in patients with PD. By synthesizing the evidence according to sleep phenotype and outcome domain, this review aims to provide a more comprehensive and clinically informative summary of the current evidence base, while highlighting the limitations that should guide interpretation and future research.

## Methods

### Protocol and registration

This systematic review and meta-analysis were conducted in accordance with the Cochrane Handbook for Systematic Reviews of Interventions and reported according to the Preferred Reporting Items for Systematic Reviews and Meta-Analyses (PRISMA) statement. The protocol was developed a priori and registered in PROSPERO (registration number: CRD420261412614).

### Eligibility criteria

Studies were selected according to the Population, Intervention, Comparator, Outcome, and Study Design framework.

Eligible studies included adults diagnosed with PD according to any validated or clinically accepted diagnostic criteria, including, but not limited to, the UK Parkinson’s Disease Society Brain Bank criteria or the International Parkinson and Movement Disorder Society criteria. Studies including participants with PD and any sleep disturbance were considered eligible, regardless of the method used to define the sleep condition, including validated sleep scales, polysomnography, actigraphy, clinical diagnosis, or self-reported sleep complaints.

Eligible interventions included any pharmacological treatment used to improve sleep disturbances in PD, either as monotherapy or in combination with another pharmacological agent. Interventions included, but were not limited to, melatonin or melatonin receptor agonists, benzodiazepines, sedative-hypnotics, dopaminergic agents, wake-promoting agents, antidepressants, antipsychotics, cholinesterase inhibitors, anticonvulsants, cannabinoids, orexin receptor antagonists, and other drug therapies. Comparators included placebo, no treatment, usual care, or another pharmacological intervention. Only RCTs were included, comprising parallel-group and crossover designs. No restrictions were applied regarding year of publication, language, country, intervention dose, treatment duration, or follow-up period.

Studies were excluded if they were observational studies, case reports, case series, narrative reviews, systematic reviews, editorials, letters without original data, animal studies, or non-randomised trials. Studies including patients with secondary or atypical parkinsonism, such as progressive supranuclear palsy or multiple system atrophy, were excluded unless data for idiopathic PD were reported separately. Conference abstracts or records without extractable data were excluded unless sufficient information could be obtained from the authors.

### Information sources and search strategy

A comprehensive literature search was conducted in PubMed/MEDLINE, Embase, the Cochrane Central Register of Controlled Trials, Web of Science, Scopus, and LILACS databases between December 2025 and April 2026. No restrictions were applied regarding publication year, language, country, intervention dose, or follow-up duration. Searches were designed to identify RCTs evaluating pharmacological interventions for sleep disturbances in patients with PD.

The search strategy was structured around three main concepts: (1) Parkinson’s disease; (2) sleep disturbances; and (3) pharmacological treatment. For each database, search terms were adapted according to the specific syntax and indexing structure of the platform. The Parkinson’s disease concept included terms such as “Parkinson Disease”, “Parkinson’s Disease”, “Parkinsonism”, and “PD”. The sleep concept included terms related to sleep disorders, sleep–wake disorders, insomnia, REM sleep behaviour disorder, restless legs syndrome, excessive daytime sleepiness, somnolence, sleep fragmentation, sleep quality, and sleep disturbances. The pharmacological intervention concept included broad terms such as drug therapy, pharmacological treatment, pharmacotherapy, medications, and pharmaceutical preparations, as well as specific drug or drug-class terms, including melatonin, ramelteon, benzodiazepines, clonazepam, zolpidem, zopiclone, eszopiclone, dopaminergic receptor agonists, pramipexole, ropinirole, rotigotine, levodopa, carbidopa, antipsychotics, quetiapine, antidepressants, trazodone, mirtazapine, doxepin, modafinil, methylphenidate, gabapentin, pregabalin, rivastigmine, donepezil, suvorexant, hypnotics, and sedatives.

Randomised trial terms and filters were applied when appropriate for each database, including terms such as “randomized”, “randomised”, “randomized controlled trial”, “controlled clinical trial”, “placebo”, “double blind”, “trial”, and “randomly”. Database-specific field tags were used where applicable, including title/abstract fields in PubMed, Embase, Scopus, and LILACS, Topic Search in Web of Science, and Trials search syntax in the Cochrane Central Register of Controlled Trials.

### Study selection

All retrieved records were imported into a reference management software, and duplicates were removed before screening. Two reviewers (L.C.S. and F.S.A.) screened titles and abstracts independently, according to the eligibility criteria. Potentially eligible studies underwent full-text review by the same two reviewers. Disagreements at any stage were resolved by consensus or by consultation with a third reviewer (J.B.C.). Reasons for exclusion at the full-text stage were recorded and summarized in the PRISMA flow diagram.

### Data extraction

Two reviewers independently (L.C.S. and F.S.A.) extracted data using a standardised data extraction form. Extracted information included study identification, publication year, country, study design, sample size, diagnostic criteria for PD, sleep disturbance phenotype, criteria or instrument used to assess sleep disturbance, participant characteristics, intervention and comparator details, dose, route of administration, treatment duration, follow-up period, outcome measures, adverse events, and funding sources.

For continuous outcomes, means, standard deviations, standard errors, confidence intervals, change-from-baseline values, and sample sizes were extracted whenever available. For dichotomous outcomes, the number of events and total number of participants per group were extracted. When relevant data were missing or unclear, study authors were contacted.

### Outcomes

The primary efficacy outcomes were sleep quality and improvement in specific sleep disturbance phenotypes, assessed using validated or clinically accepted instruments. Primary outcomes included, depending on the sleep phenotype, Pittsburgh Sleep Quality Index (PSQI) scores, Parkinson’s Disease Sleep Scale scores (PDSS), Epworth Sleepiness Scale (ESS) scores, Insomnia Severity Index (ISI) scores, RBD frequency or severity, and polysomnographic or actigraphic sleep parameters.

The PDSS and its revised version, the PDSS-2, are related but distinct instruments and were not used interchangeably in this review: the original PDSS was used for the overall nocturnal sleep/insomnia symptom domain, whereas the PDSS-2, a psychometrically revised, 15-item scale with a different item structure, was used as a proxy severity measure in the RBD domain, in the absence of a dedicated, validated RBD severity instrument in the contributing trials. Pooled estimates derived from the PDSS and the PDSS-2 are therefore not directly comparable to one another. Secondary outcomes included adverse events, serious adverse events, treatment discontinuation due to adverse events, quality of life, motor symptoms, non-motor symptoms, total sleep time, sleep onset latency, wake after sleep onset, sleep efficiency, apnea–hypopnea index, and other sleep-related outcomes reported in the included trials. Whenever possible, outcomes were grouped by sleep disturbance phenotype, including insomnia symptoms, EDS, RBD, restless legs syndrome, poor sleep quality, and non-specific sleep disturbances.

### Risk of bias assessment

The risk of bias of included RCTs was assessed independently by two reviewers (L.C.S. and F.S.A.) using the Cochrane Risk of Bias 2 tool (RoB 2). Parallel-group trials were assessed using the standard RoB 2 tool for individually randomised, parallel-group trials, whereas crossover trials were assessed using the RoB 2 tool specifically adapted for crossover trials, which incorporates additional signalling questions addressing carry-over effects, period effects, and the adequacy of washout. The certainty of evidence for each primary outcome was assessed using the Grading of Recommendations Assessment, Development and Evaluation (GRADE) framework, considering risk of bias, inconsistency, imprecision, indirectness, and publication bias. The assessment considered bias arising from the randomisation process, deviations from intended interventions, missing outcome data, measurement of outcomes, and selection of reported results. For crossover trials, additional considerations related to carry-over effects, period effects, and adequacy of washout were examined when applicable. Disagreements were resolved by consensus or by consultation with a third reviewer (J.B.C.).

### Statistical analysis

All statistical analyses were performed using custom Python scripts (version 3.11; NumPy, pandas, SciPy, and Matplotlib libraries) implementing algorithms in strict accordance with the recommendations of the Cochrane Handbook for Systematic Reviews of Interventions [18]. Effect sizes were expressed as the mean difference (MD) or weighted mean difference (WMD) with 95% confidence intervals (CIs) when all studies within a given domain used the same scale and units; the standardised mean difference (SMD; Hedges' g) was reserved for instances where included studies employed scales with different metrics for the same clinical construct. For all outcomes, the direction of the effect was aligned so that a positive MD or SMD indicates clinical improvement, in accordance with the respective scale conventions.

When primary studies reported means and standard deviations (SDs) at baseline and endpoint but did not provide change-from-baseline data directly, the mean change was computed as the arithmetic difference between endpoint and baseline means. The SD of the change score was imputed using the formula SD₁₂ = √(SD^2^pre + SD^2^post − 2r·SDpre·SDpost), where r denotes the pre–post correlation coefficient, as recommended by the Cochrane Handbook [18, 19]. A correlation coefficient of r = 0.80 was applied as the primary value, consistent with estimates reported in prior meta-analyses on sleep outcomes in neurodegenerative disease [17]. To evaluate the robustness of this assumption, a pre-specified sensitivity analysis was conducted by repeating all pooled estimates with r = 0.50, r = 0.70, and r = 0.90.

For crossover trials, a paired standard error was applied rather than the conventional independent-groups formula, in line with the analytical approach specified for within-subject designs in the Cochrane Handbook (Sect.  23.2.2) [18]. The paired SE was calculated as SE = SD_paired/√n, where SD_paired = √(SD^2^A + SD^2^B − 2r·SDA·SDB), with SDA and SDB denoting the SDs of the change scores for the active and control periods, respectively. When the SD of the change score for each treatment period was not reported explicitly, it was imputed from the period-specific pre- and post-treatment SDs using the formula described above.

One included three-arm trial [20] contributed two independent pairwise comparisons to the meta-analysis of excessive daytime sleepiness. To avoid double-counting of participants in the shared control arm, the control group sample size was split between the two comparisons (n₁ = 6 and n₂ = 5), with means and SDs retained as reported, following the approach recommended for multi-arm trials in the Cochrane Handbook (Sect.  23.3.4) [18]. No formal statistical adjustment for multiple comparisons was applied across the two pairwise comparisons derived from this trial, as both were treated as hypothesis-generating components of the same pre-specified random-effects model rather than as independent confirmatory tests.

Pooling was performed using the DerSimonian–Laird random-effects model [19], which accounts for both within-study sampling variance and between-study variance (τ^2^). A random-effects model was selected a priori given the anticipated clinical and methodological heterogeneity arising from the diversity of pharmacological interventions, outcome instruments, treatment durations, and patient populations. Statistical significance was set at α = 0.05.

Statistical heterogeneity was quantified using the I^2^ statistic, Cochran's Q test, and τ^2^. I^2^ values were interpreted as follows: below 25% as low, 25–49% as moderate, 50–74% as substantial, and ≥ 75% as considerable heterogeneity. Significance of heterogeneity was assessed at p < 0.10 [18]. When outcomes could not be combined quantitatively owing to an insufficient number of eligible studies (k < 2) or substantial clinical heterogeneity, results were synthesized narratively.

Pre-specified subgroup analyses were conducted for the two domains with the largest number of eligible studies to explore potential sources of heterogeneity, stratified by: (1) pharmacological class; and (2) treatment duration (≤ 4 weeks vs. > 4 weeks). Study-level weights in subgroup forest plots were expressed as proportions of the total across all strata, using subgroup-specific τ^2^, following RevMan conventions [22]. Although these subgroup comparisons were specified a priori in the registered protocol, we acknowledge that, for the EDS domain, the dopaminergic receptor agonist and chronobiotic subgroups each comprised a single trial; estimates from these single-trial subgroups are therefore likely to be underpowered and are reported as hypothesis-generating rather than confirmatory.

Four pre-specified sensitivity analyses were performed: (1) leave-one-out analysis for each domain; (2) variation of the imputation correlation r (0.50, 0.70, 0.90); (3) exclusion of a crossover trial [41] in which period-specific baseline values were assumed equal in the absence of reported data; and (4) inclusion of the active-comparator trial [21] in the sleep quality domain. Leave-one-out results for all four outcome domains are consolidated in Table 4.

Publication bias was assessed using contour-enhanced funnel plots and Egger's linear regression test for domains with at least ten eligible studies, in accordance with the Cochrane Handbook (Sect. 13.1.2) [18]. Formal testing was not performed for domains with fewer than ten studies; this is noted explicitly in the respective forest plot legends.

## Results

**Fig. 1:**
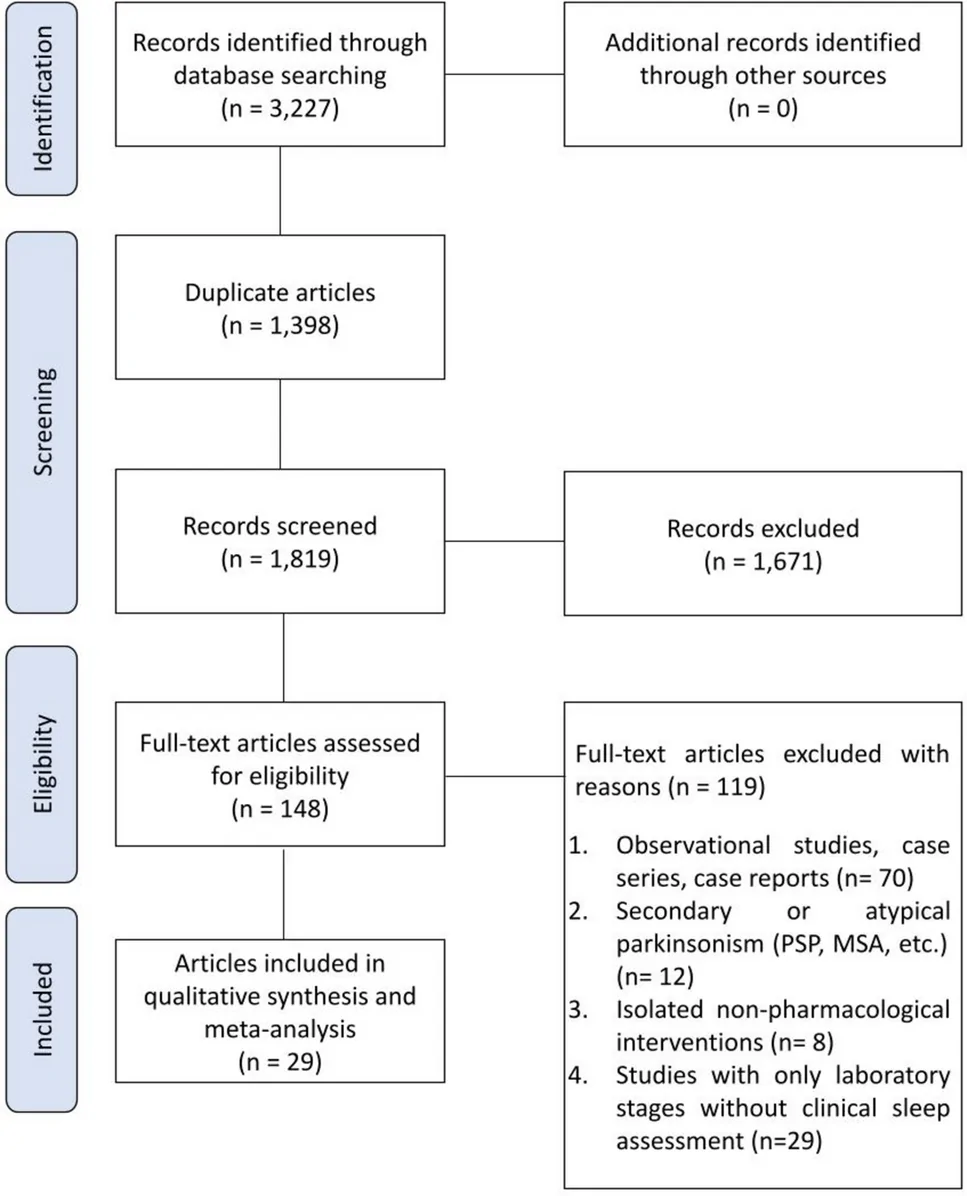
The PRISMA flow chart

A total of 3227 studies were identified through database searching. Of these, 148 records underwent full-text review, of which 119 were excluded. Twenty-nine RCTs met all eligibility criteria and were included in the quantitative synthesis (Fig. 1).

### Characteristics of included studies

The 29 included RCTs enrolled a total of 1913 participants across studies published between 2002 and 2025. The trials were conducted in 14 countries across five continents, with Europe contributing the largest proportion of studies (n = 11; including France, Germany, Italy, Austria, and Switzerland), followed by Asia (n = 7; Iran, Japan, South Korea, and China), North America (n = 6; USA and Canada), South America (n = 2; Brazil), South Asia (n = 1; India), and Oceania (n = 1; Australia). One study was multinational [23]. Across the 22 trials reporting age, the mean age of participants ranged from 56 years [24] to 76 years [25], reflecting the predominantly older adult PD population. The proportion of male participants ranged widely, from 20% [24] to 89% [26], with most trials reporting a male predominance consistent with the known epidemiology of PD. The full characteristics of each included study are presented in Table 1.

**Table 1:** Baseline characteristics of randomised controlled trials included in the systematic review and meta-analysis

Author (Year)	Country	N (I/C)	Age, mean ± SD (years)	Male (%)	PD Duration, mean ± SD (years)	H&Y Stage	UPDRS-III, mean ± SD	LEDD, mean ± SD (mg/day)	PD Diagnostic Criteria
Adler et al. (2002) [28]	USA	20 (11/10)	65 ± 12	70%	7.4 ± 4.9	2.0 ± 0.5	14.4 ± 8.5	830 ± 420	Idiopathic PD (NOS)
Ahn et al. (2020) [44]	South Korea	34 (16/18)	PRM: 66.0 ± 7.5 Placebo: 64.6 ± 6.5	50%	PRM: 5.0 ± 5.9 Placebo: 4.2 ± 4.4	1.7 ± 0.5–0.8	15.2 (Part III)	NR	UK Brain Bank + PET
Almeida et al. (2021) [36]	Brazil	33 (17/16)	57 (overall)	NR	NR	NR	NR	NR	UK Brain Bank
Antonini et al. (2015) [23]	Multinational	349 (224/125)	67.5 ± 9.6	56.2%	NR	Stages I–IV	~26 (Part III)	69.3% on levodopa	NR
Büchele et al. (2017) [29]	Switzerland	12 (6/6)	62 ± 11.1	83.3%	8.4 ± 4.6	II or III	NR	775 ± 497	NR
Chitsaz et al. (2024) [20]	Iran	59 (22/26/11)	65.7–71.5 (group means)	63–82% (per arm)	4.9–8.0 (per arm)	NR	NR	NR	NR
Corvol et al. (2022) [30]	France	75 (crossover)	63.5 ± 9.4	67%	8.6 ± 5.3	≤4	NR	781 ± 484	MDS criteria
de Cock et al. (2022) [43]	France	46 (44/44)	63.6 ± 9.2	60%	9.8 ± 4.5	NR	16.2–16.4 (MDS Part II)	NR	Queen Square Bank
Dowling et al. (2005) [27]	USA	40 (crossover)	61.7 ± 8.4	72.5%	7.5 ± 4.2	2.4 ± 0.6	23.5 ± 13.6	282 ± 192	Idiopathic PD
Eggert et al. (2014) [31]	Germany	68 (36/32)	NR	NR	NR	NR	NR	NR	NR
Gilat et al. (2020) [37]	Australia	30 (15/15)	NR	NR	NR	NR	NR	NR	UK Brain Bank
Hadi et al. (2022) [21]	Iran	112 (31/31/31)	67.19 ± 8.26 (melatonin arm)	65–71% (per arm)	5.06–5.57 ± 4.97–5.19	1–2 (median)	NR	NR	UK Brain Bank
Högl et al. (2002) [32]	Austria	15 (crossover)	65.0 ± 7.6	75%	6.8 ± 4.1	Median 2.3	NR	591 ± 219 (levodopa)	UK Brain Bank
Kakhaki et al. (2020) [38]	Iran	60 (25/26)	Mel: 64.4 ± 8.2 Pbo: 66.3 ± 9.3	Mel: 64% Pbo: 61.5%	Mel: 5.7 ± 1.9 Pbo: 5.5 ± 2.1	NR	Mel: 73.6 ± 26.9 Pbo: 70.3 ± 21.4 (total)	Mel: 597.5 ± 195.0 Pbo: 596.2 ± 204.6	UK Brain Bank
Kataoka et al. (2024) [39]	Japan	69 (34/35)	66–68 (per arm)	36.4–47.1%	NR	Stages I–IV	Zoni: 27.9 ± 15.0 Pbo: 23.9 ± 13.1	NR	MDS criteria
Medeiros et al. (2007) [26]	Brazil	18 (8/10)	61.80 ± 7.13	88.9%	7.05 ± 4.87	Stages I–III	16–17 (Parts II–III)	600–650 (levodopa)	Clinical criteria
Meloni et al. (2022) [40]	Italy	18 (16/16)	67.5 ± 7.4	66.7%	8.1 ± 4.7	NR	30.6 ± 18.8	728.6 ± 509.1	UK Brain Bank
Menza et al. (2010) [24]	USA	30 (15/15)	56	20%	4.5	1.6	26.7 ± 9.8	NR	Research criteria
Ondo et al. (2005) [33]	USA	40 (20/20)	64.8 ± 11.3	72.5%	6.8 ± 5.0	NR	26.7 ± 9.8 ('on')	NR	NR
Pierantozzi et al. (2016) [45]	Italy	42 (21/21)	Rot: 63.28 ± 2.98 Pbo: 64.04 ± 2.90	NR	~50 months	Stages 2–3	26.1–26.8 ± 12.1–12.6	478–505 (levodopa)	UK Brain Bank
Plastino et al. (2021) [41]	Italy	30 (15/15)	A+: 66 ± 7.2 B−: 64 ± 8.7	60%	5.7–6.3	2.5–3.0	NR	NR	UK Brain Bank
Postuma et al. (2012) [34]	Canada/Brazil	61 (30/31)	Caf: 65.5 ± 9.1 Pbo: 66.8 ± 11.2	Caf: 83.3% Pbo: 61.3%	Caf: 8.4 ± 5.0 Pbo: 8.4 ± 4.5	NR	Caf: 42.0 ± 17.5 Pbo: 41.2 ± 13.1	Caf: 679.5 ± 505.7 Pbo: 662.3 ± 461.3	Idiopathic PD
Ricciardi et al. (2015) [25]	Italy	47 (25/22)	Hom: 76.1 ± 5.7 Ctrl: 69.6 ± 9.0	81% (intervention)	~12–13	NR	~25–32	~770–815	NR
Schrempf et al. (2018) [46]	Germany	30 (20/10)	65.5 ± 7.9	65%	4.8 ± 4.4	1.9 ± 0.8	Clo: 23.4 ± 10.4 Pbo: 24.3 ± 8.8	344 ± 308	UK Brain Bank
Shin et al. (2019) [42]	South Korea	39 (19/20)	Clo: 66.0 (48–73) Pbo: 70.0 (56–77)	Clo: 52.6% Pbo: 45.0%	Clo: 6.0 Pbo: 8.0 (median)	Clo: 2.0 Pbo: 2.5 (median)	Clo: 23.4 ± 10.4 Pbo: 24.3 ± 8.8	Clo: 1000 Pbo: 675 (median)	UK Brain Bank
Sugumaran et al. (2024) [47]	India	73 (35/38)	Mel: 60.3 ± 6.3 Pbo: 58.4 ± 8.8	~62%	Mel: 3.0 ± 1.8 Pbo: 3.6 ± 3.6	1.8 ± 0.6	57.6 ± 10.6 (total)	Mel: 506.0 ± 155.8 Pbo: 478.6 ± 135.0	UK Brain Bank
Videnovic et al. (2021) [35]	USA	64 (crossover)	NR	NR	NR	NR	NR	NR	NR
Wu et al. (2025) [48]	China	55 (28/27)	NHD: 64.29 ± 6.44 Pbo: 68.25 ± 8.53	NHD: 42.9% Pbo: 44.4%	NHD: 3.77 ± 3.26 Pbo: 4.65 ± 6.12	≤3	NHD: 27.5 (14.0) Pbo: 30.0 (16.0)	400 (median)	MDS 2015
Zhang et al. (2013) [49]	China	344 (175/169)	NR	NR	NR	NR	Rot: 28.8 ± 13.3 Pbo: 29.3 ± 12.3	NR	NR

Unless otherwise specified, values are reported as mean ± standard deviation (SD). Age and PD duration reported as available from each study; some values are approximate or represent the full sample (not per arm). H&Y values are reported as mean ± SD or median (range) as originally reported. LEDD values represent levodopa equivalent daily dose or equivalent dopaminergic dose. Years shown for Adler et al. and Büchele et al. correspond to first online publication (print: 2003 and 2018, respectively).

*Caf* caffeine, *Clo* clonazepam, *H*&*Y* Hoehn and Yahr scale, *Hom* homotaurine, *I* intervention group, *LEDD* levodopa equivalent daily dose, *Mel* melatonin, *MDS* Movement Disorder Society, *NHD* nyctinastic herbs decoction, *NOS* not otherwise specified, *NR* not reported, *Pbo* placebo, *PD* Parkinson's disease, *PRM* prolonged-release melatonin, *Rot* rotigotine, *UPDRS*-*III* Unified Parkinson's Disease Rating Scale Part III (motor)

Mean disease duration, reported in 20 trials, ranged from 3 to 13 years. Most participants were in mild-to-moderate stages of PD, with Hoehn and Yahr (H&Y) scores predominantly between 1.5 and 3 in the trials that reported this measure. Baseline motor severity, assessed by the Unified Parkinson's Disease Rating Scale Part III (UPDRS-III), ranged from 14 to 58 points across studies, indicating a broad spectrum of motor impairment. Levodopa equivalent daily dose (LEDD), reported in 15 trials, ranged from 282 mg/day [27] to 830 mg/day [28], reflecting the variability in concomitant dopaminergic treatment across study populations.

The PD diagnosis was most established using the United Kingdom Brain Bank Criteria (n = 15 trials), followed by the Movement Disorder Society (MDS) criteria (n = 4). Two studies used criteria not otherwise specified [27, 28], and the remaining trials applied clinical or institutional diagnostic criteria. Sleep disorder diagnosis at inclusion was based on validated rating scales in most trials, including the ESS, PSQI, video-polysomnography or the RBD Screening Questionnaire (RBDSQ; for RBD), ISI, or PDSS/PDSS-2. Stable concomitant antiparkinsonian therapy was required as an inclusion criterion in most trials, and most studies explicitly prohibited the concurrent use of sedative or hypnotic medications during the study period.

The included trials addressed four main sleep disorder domains. Ten trials focused on EDS [20, 25, 28–35]. Seven trials investigated RBD [36–42]. Two trials focused on insomnia symptoms [24, 43]. The remaining ten trials examined overall sleep quality or nocturnal sleep disturbances [21, 23, 26, 27, 44–49].

Pharmacological interventions spanned multiple drug classes (Table 2). Chronobiotic agents (melatonin and prolonged-release melatonin) were the most frequently evaluated, investigated in seven trials [21, 26, 27, 37, 38, 44, 47] at doses ranging from 2 to 10 mg. Wake-promoting agents and psychostimulants were evaluated in seven trials, including modafinil [20, 28, 32, 33], methylphenidate [20], THN102, a fixed-dose combination of modafinil and low-dose flecainide [30], solriamfetol [35], and sodium oxybate [29]. Popaminergic receptor agonists were examined in five trials: rotigotine transdermal patch [23, 45], apomorphine subcutaneous infusion [43], piribedil [31], and ropinirole prolonged-release [49]. MAO-B inhibitors were evaluated in two trials: safinamide [41] and rasagiline [46]. Additional pharmacological agents evaluated in single trials included cannabidiol [36], zonisamide [39], clonazepam [21, 42], eszopiclone [24], trazodone [21], 5-hydroxytryptophan [40], caffeine [34], homotaurine [25], and a nyctinastic herbs decoction [48].

**Table 2:** Characteristics of pharmacological interventions and sleep-related outcomes in the randomised controlled trials included in the systematic review

Author (Year)	Country	Study design	Sleep disorder	N (I/C)	Drug/dose	Comparator	Duration	Primary outcome	Key sleep finding	Funding
Adler et al. (2002) [28]	USA	RCT, DB, crossover	EDS	20 (11/10)	Modafinil 200 mg/day	Placebo	3 weeks	ESS	Modafinil improved subjective EDS; no significant PSG change	Cephalon
Ahn et al. (2020) [44]	South Korea	RCT, DB, parallel	Poor sleep quality	34 (16/18)	Melatonin PRM 2 mg	Placebo	4 weeks	PSQI	Significant PSQI improvement vs. placebo	Kuhnil Pharma
Almeida et al. (2021) [36]	Brazil	RCT, DB, parallel	RBD	33 (17/16)	Cannabidiol 75–300 mg/day	Placebo	12 weeks	PSG/sleep diary	No significant reduction in RBD episodes	INCT-TM/CNPq
Antonini et al. (2015) [23]	Multinational	RCT, DB, parallel	Sleep/fatigue (NMS)	349 (224/125)	Rotigotine 8–16 mg/24 h (patch)	Placebo	7 weeks	NMSS (Domain 2)	Both groups improved similarly; no significant advantage for rotigotine	NR
Büchele et al. (2017) [29]	Switzerland	RCT, DB, crossover	EDS + nocturnal sleep	12 (6/6)	Sodium oxybate 4.8 g	Placebo	6 weeks	ESS/MSLT	Significant improvement in EDS and nocturnal sleep	CB Pharma
Chitsaz et al. (2024) [20]	Iran	RCT, DB, 3-arm	EDS	59 (22/26/11)	Modafinil 200 mg/day; Methylphenidate 10 mg/day	Placebo	6 weeks	ESS/PSQI	Both drugs improved EDS and sleep quality; no difference between them	Isfahan Univ
Corvol et al. (2022) [30]	France	RCT, DB, 3-way crossover (Ph.2a)	EDS	75 (active/placebo crossover)	THN102 200/2 mg; 200/18 mg (modafinil + flecainide)	Placebo	2 weeks/arm	ESS	THN102 200/2 mg showed efficacy signal; well tolerated	Theranexus
de Cock et al. (2022) [43]	France	RCT, DB, crossover	insomnia symptoms	46 (44/44)	Apomorphine SC infusion (nocturnal)	Placebo infusion	4 weeks/arm	PDSS/ISI	Significant improvement in sleep efficiency and PDSS	NR
Dowling et al. (2005) [27]	USA	RCT, DB, crossover	Nocturnal sleep	40	Melatonin 5 mg or 50 mg	Placebo	2 weeks/arm	Sleep time (actigraphy)	No significant improvement in ESS or sleep time	NR
Eggert et al. (2014) [31]	Germany	RCT, active comparator	EDS	68 (36/32)	Piribedil 100–300 mg/day	Pramipexol or ropinirole	11 weeks	ESS	No significant difference in vigilance between piribedil and comparators	Desitin GmbH
Gilat et al. (2020) [37]	Australia	RCT, DB, parallel	RBD	30 (15/15)	Melatonin PRM 4 mg	Placebo	8 weeks	RBD episodes (v-PSG)	No significant reduction in RBD episodes vs. placebo	NR
Hadi et al. (2022) [21]	Iran	RCT, DB, 3-arm	Sleep complaints	112 (31/31/31)	Melatonin 3 mg; Trazodone 50 mg	Clonazepam 1 mg	4 weeks	PSQI/ESS/RBDSQ	All arms improved; melatonin superior on RBDSQ; trazodone superior on ESS vs. clonazepam	NR
Högl et al. (2002) [32]	Austria	RCT, DB, crossover	EDS	15 (crossover)	Modafinil 100–200 mg/day	Placebo	2 weeks/arm	ESS/PSG	Significant ESS improvement; no PSG change	NR
Kakhaki et al. (2020) [38]	Iran	RCT, DB, parallel	RBD/RLS	60 (25/26)	Melatonin 10 mg	Placebo	12 weeks	PSQI	Significant PSQI improvement and metabolic/inflammatory benefits	Kashan Univ
Kataoka et al. (2024) [39]	Japan	RCT, SB, parallel (ZEAL)	RBD	69 (34/35)	Zonisamide 25 mg	Placebo	28 days	PDSS-2/RBDSQ	No significant improvement in sleep outcomes vs. placebo	Sumitomo Pharma
Medeiros et al. (2007) [26]	Brazil	RCT, DB, parallel	Poor sleep quality	18 (8/10)	Melatonin 3 mg	Placebo	4 weeks	PSQI	Significant PSQI improvement vs. placebo	NR
Meloni et al. (2022) [40]	Italy	RCT, DB, crossover	RBD	18 (16/16)	5-Hydroxytryptophan (5-HTP)	Placebo	4 weeks/arm	PSG (REM sleep %)	Possible increase in REM sleep percentage; preliminary finding	Sleep Disorders Ctr
Menza et al. (2010) [24]	USA	RCT, DB, parallel	insomnia symptoms	30 (15/15)	Eszopiclone 2–3 mg	Placebo	6 weeks	TST/awakenings	Significant reduction in nocturnal awakenings; no improvement in TST	Sepracor
Ondo et al. (2005) [33]	USA	RCT, DB, parallel	EDS	40 (20/20)	Modafinil 200–400 mg/day	Placebo	4 weeks	ESS	No significant ESS improvement vs. placebo	Cephalon
Pierantozzi et al. (2016) [45]	Italy	RCT, DB, parallel	Sleep quality + architecture	42 (21/21)	Rotigotine 16 mg/24 h (patch)	Placebo	10 days	PSQI/PDSS/PSG	Significant improvement in PSQI, sleep efficiency, and REM sleep	NR
Plastino et al. (2021) [41]	Italy	RCT, crossover (pilot)	RBD	30 (15/15)	Safinamide 50 mg	UAT (without safinamide)	3 months/arm	PDSS-2	Significant reduction in PDSS-2; improved RBD symptoms and motor function	NR
Postuma et al. (2012) [34]	Canada/Brazil	RCT, parallel	EDS	61 (30/31)	Caffeine 100–200 mg BID	Placebo	6 weeks	ESS	Equivocal ESS benefit; significant improvement in CGI-C	NR
Ricciardi et al. (2015) [25]	Italy	RCT, parallel	EDS	47 (11/13 analysed)	Homotaurine 100 mg	Control	12 weeks	ESS	Numerically improved UPDRS-I and ESS in homotaurine arm	NR
Schrempf et al. (2018) [46]	Germany	RCT, DB, baseline-controlled	Sleep quality	30 (20/10)	Rasagiline 1 mg/day	Placebo	8 weeks	PDSS-2/PSG	Improved PSG parameters (WASO, sleep maintenance) and ESS	NR
Shin et al. (2019) [42]	South Korea	RCT, DB, parallel	Probable RBD	39 (19/20)	Clonazepam 0.5 mg	Placebo	4 weeks	RBD1Q/CGI	No significant between-group difference; trend toward improvement in both arms	NR
Sugumaran et al. (2024) [47]	India	RCT, DB, parallel	Poor sleep quality	73 (35/38)	Melatonin 3 mg	Placebo	8 weeks	PSQI/ESS	Significant improvement in PSQI, ESS, and objective sleep parameters	NR
Videnovic et al. (2021) [35]	USA	RCT, DB, crossover (Ph.2)	EDS	64 (crossover)	Solriamfetol 75–300 mg/day	Placebo	4 weeks/arm	ESS/MWT	No significant ESS improvement; possible benefit on MWT	Jazz Pharma
Wu et al. (2025) [48]	China	RCT, DB, parallel	Sleep–wake rhythm disorder	55 (28/27)	Nyctinastic herbs decoction (NHD)	Placebo	12 weeks	RBDQ-HK/actigraphy	Significant improvement in SWRD symptoms and sleep fragmentation	Shanghai Health Comm
Zhang et al. (2013) [49]	China	RCT, DB, parallel, multicenter	Sleep quality (advanced PD)	344 (175/169)	Ropinirole PR (titrated)	Placebo	24 weeks	PDSS/motor 'off' time	Significant reduction in total 'off' time; PDSS did not improve significantly	GlaxoSmithKline

Years shown for Adler et al. and Büchele et al. correspond to first online publication (print: 2003 and 2018, respectively)

*DB* double-blind, *EDS* excessive daytime sleepiness, *ESS* Epworth Sleepiness Scale, *H*&*Y* Hoehn and Yahr scale, *I* intervention group, *ISI* Insomnia Severity Index, *MDS* Movement Disorder Society, *MSLT* Multiple Sleep Latency Test, *NMS* Non-motor symptoms, *NMSS* Non-Motor Symptoms Scale, *NOS* not otherwise specified, *NR* Not reported, *PD* Parkinson's disease, *PDSS* Parkinson's Disease Sleep Scale, *Ph* phase, *PRM* prolonged-release melatonin, *PSG* polysomnography, *RBD* rapid eye movement sleep behaviour disorder, *RBDQ*-*HK RBD* Questionnaire Hong Kong, *RCT* randomised controlled trial, *REM* rapid eye movement, *SB* single-blind, *SC* subcutaneous, *SWRD* sleep–wake rhythm disorder, *TST* Total sleep time, *UAT* usual antiparkinsonian therapy, *UK* United Kingdom, *v*-*PSG* video-polysomnography, *WASO* wake after sleep onset

### Risk of bias assessment

**Fig. 2:**
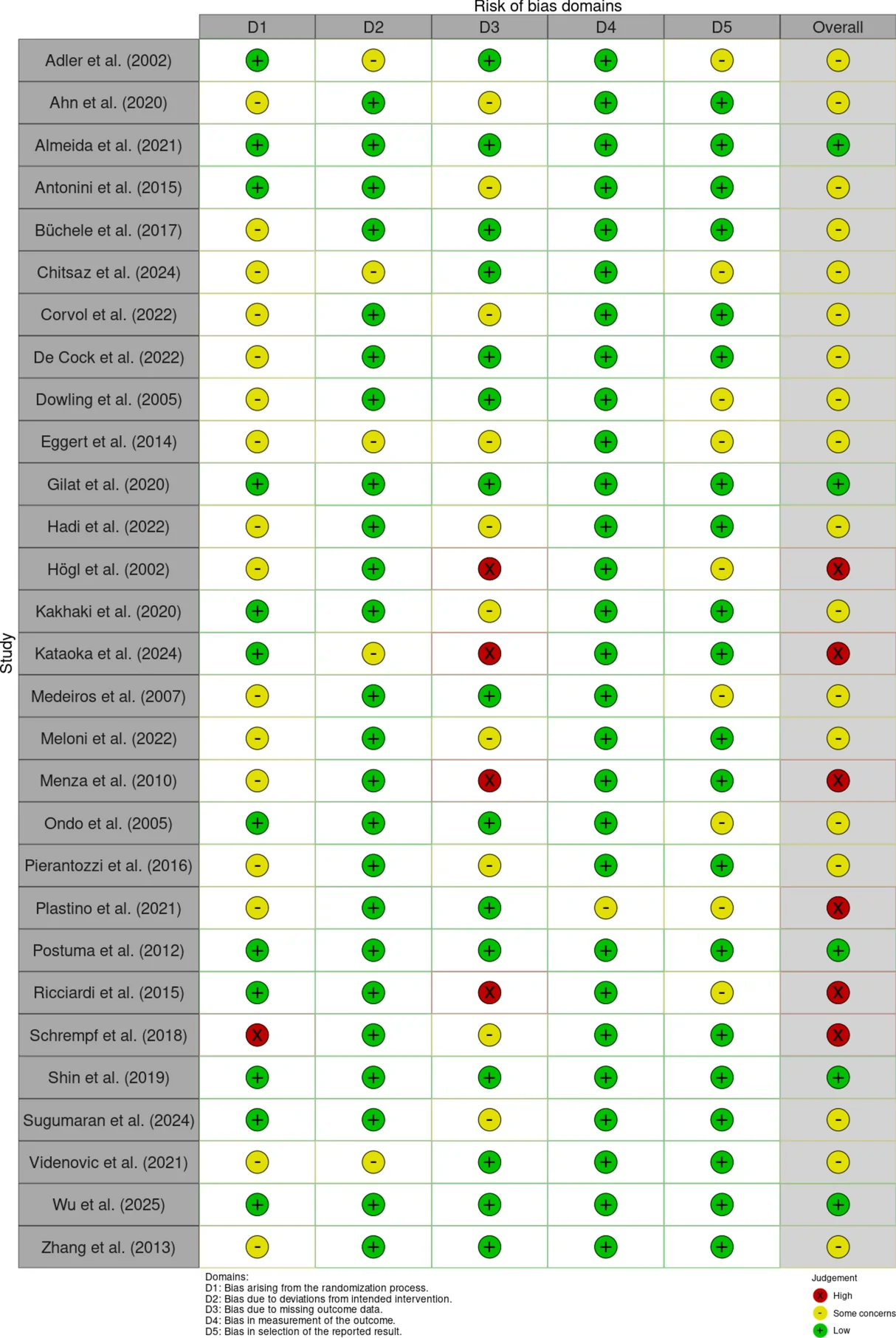
Study-level risk-of-bias assessment using the RoB 2 tool

The study-level judgments are shown in the traffic-light plot, and the proportional distribution of judgments across domains is summarized in Fig. 2. Overall, only 5 of 29 trials (17.2%) were judged to have low risk of bias, whereas 18 trials (62.1%) raised some concerns and 6 trials (20.7%) were classified as having high risk of bias. The main sources of concern were related to the randomization process and missing outcome data. For the randomization domain, 12 trials were rated as low risk, 16 as having some concerns, and one trial as high risk, suggesting that allocation procedures, concealment, or baseline comparability were not consistently reported or fully reassuring. Missing outcome data was the most problematic domain: 15 trials were rated as low risk, 10 as having some concerns, and 4 as high risk, indicating that attrition, incomplete follow-up, or insufficient handling of missingness may have affected the certainty of some estimates.

By contrast, bias due to deviations from intended interventions and outcome measurement appeared less problematic. Most trials were judged as low risk for deviations from intended interventions, with 24 of 29 studies classified as low risk and no study judged as high risk in this domain. Similarly, outcome measurement was generally robust, with 28 studies rated as low risk and only one study raised some concerns. Bias in the selection of the reported result was also not classified as high risk in any trial; however, 9 studies raised some concerns, mainly reflecting incomplete information regarding prespecified outcomes, analysis plans, or selective reporting safeguards. Overall, with only 17.2% of trials judged at low risk of bias, confidence in the direction and magnitude of all pooled estimates reported below is substantially limited, irrespective of statistical significance, and this caveat should be borne in mind throughout the interpretation of the meta-analytic findings.

The certainty of evidence for each outcome domain was assessed using the GRADE framework and is summarised in Table 3. A summary of the sensitivity analyses using the “leave-one-out” method across the different outcome domains is presented in Table 4.

**Table 3:** Summary of Findings—GRADE Assessment of certainty of evidence for pharmacological interventions for sleep disorder phenotypes in Parkinson's disease

Outcome	Studies (k)	Participants (n)	Pooled effect (MD; 95% CI)	Risk of bias	Inconsistency	Imprecision	Indirectness	Publication bias	Certainty
Excessive daytime sleepiness (ESS)	6 arms, 5 RCTs	261	MD 1.75 (− 0.26 to 3.75)	Serious ^1^	Very serious ^2^	Serious ^3^	Serious ^4^	Not assessable	⊕◯◯◯ VERY LOW
Overall sleep quality (PSQI)	6 RCTs	251	MD 2.66 (1.63 to 3.69)	Serious ^1^	Serious ^5^	Serious ^3^	Serious ^4^	Not assessable	⊕⊕◯◯ LOW
Insomnia symptoms (PDSS)	3 RCTs	432	MD 10.29 (− 0.58 to 21.17)	Serious ^1^	Very serious ⁶	Very serious ⁷	Serious ^4^	Not assessable	⊕◯◯◯ VERY LOW
REM sleep disorder (PDSS-2)	2 RCTs	94	MD 3.26 (− 8.11 to 14.63)	Serious ^1^	Very serious ⁸	Very serious ⁷	Serious ^4^	Not assessable	⊕◯◯◯ VERY LOW

^1^Only 17.2% of included trials were judged at low overall risk of bias (RoB 2); the majority raised some concerns or were classified as high risk, primarily due to limitations in randomisation reporting and missing outcome data. Rated down one level

^2^I^2^ = 91%; heterogeneity was high and driven by a single crossover trial with probable carry-over effects (Högl et al., 2002) producing a directionally opposite estimate to all other arms. Rated down two levels

^3^Small total sample size; wide confidence intervals; few trials per outcome (k ≤ 6). Rated down one level

^4^Heterogeneous populations, diverse pharmacological classes, variable treatment durations (2–24 weeks), and mixed outcome instruments limit direct applicability. Rated down one level

^5^I^2^ = 71%; substantial heterogeneity across drug classes and populations, though direction of effect was consistent across all six trials. Rated down one level

⁶I^2^ = 96%; high heterogeneity across three trials evaluating pharmacologically distinct agents in different populations and disease stages. Rated down two levels

⁷Very wide confidence intervals crossing zero; k = 2 or k = 3 only; pooled estimate highly sensitive to removal of individual studies. Rated down two levels

⁸I^2^ = 99%; two trials produced directionally opposite estimates, rendering the pooled estimate clinically uninformative. Rated down two levels

Certainty of evidence: ⊕⊕⊕⊕ High |⊕ ⊕⊕◯ Moderate |⊕ ⊕◯◯ Low |⊕ ◯◯◯ Very low

Interpretation: Pharmacological treatment may improve overall sleep quality in PD (low certainty), but evidence for excessive daytime sleepiness, insomnia, and REM sleep behaviour disorder is of very low certainty, meaning current evidence is insufficient to support definitive clinical recommendations

Nominally significant subgroup estimates identified within the ESS domain (dopaminergic receptor agonists and chronobiotics; Sect. "ESS") each derive from a single trial and do not upgrade the overall very low certainty rating shown above, which reflects the imprecision and inconsistency of the pooled ESS evidence base as a whole. For all three domains with I^2^ > 90% (ESS, insomnia symptoms, RBD), pooled estimates are presented as exploratory only, and narrative synthesis should be regarded as the primary basis for interpretation

*CI* confidence interval, *ESS* Epworth Sleepiness Scale, *MD* mean difference, *PDSS* Parkinson’s Disease Sleep Scale, *PDSS*-*2* Parkinson's Disease Sleep Scale version 2, *PSQI* Pittsburgh Sleep Quality Index, *RBD* REM sleep behaviour disorder, *RCT* randomised controlled trial

**Table 4:** Summary of leave-one-out sensitivity analyses across outcome domains

Outcome domain	Studies (k)	Leave-one-out MD range (95% CI)	Most influential study	Interpretation
Excessive daytime sleepiness (ESS)	6 arms, 5 RCTs	0.88 to 1.99 (all CIs cross zero), except MD 2.51 (1.02 to 3.99) on omission of one trial	Högl et al. (2002) [32]. Sole omission yielding statistical significance	Non-significant pooled result is driven by a single influential crossover trial; not robust
Overall sleep quality (PSQI)	6 RCTs	2.20 (1.48 to 2.92) to 3.11 (2.27 to 3.95), all CIs exclude the null	None disproportionately influential	Highly robust to removal of any individual trial
Insomnia symptoms (PDSS)	3 RCTs	Statistical significance achieved only on omission of one trial	Pierantozzi et al. (2016) [45]. Result then driven by the weight of Zhang et al. (2013) [49]	Pooled estimate is unstable and not robust
RBD (PDSS-2)	2 RCTs	Not performed (below minimum k = 3 threshold)	Not applicable	Insufficient studies for sensitivity testing

*CI* confidence interval, *MD* mean difference, *PDSS* Parkinson's Disease Sleep Scale, *PDSS*-*2* Parkinson's Disease Sleep Scale version 2, *PSQI* Pittsburgh Sleep Quality Index, *RBD* REM sleep behaviour disorder, *RCT* randomised controlled trial

### Meta-analysis

#### ESS

**Fig. 3:**
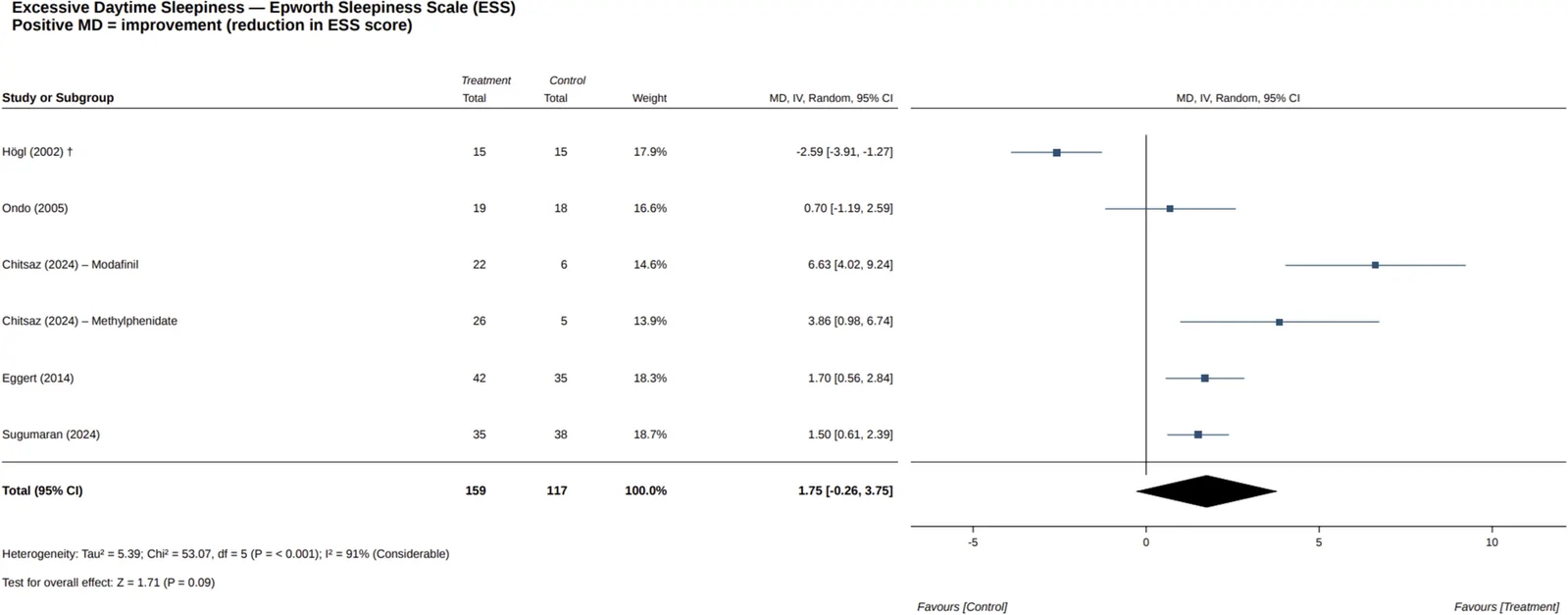
Forest plot for EDS/ESS (pharmacological treatment vs. placebo). *Positive MD* improvement [reduction in ESS score], random-effects model, inverse-variance

Six study arms from five RCTs contributed to the primary meta-analysis of EDS, measured with the ESS (Fig. 3). The included trials evaluated modafinil [32, 33], modafinil and methylphenidate [20], and melatonin [47]. The pooled mean difference (MD) was 1.75 (95% CI −0.26 to 3.75; P = 0.09; Z = 1.71), reflecting a direction of benefit in favour of pharmacological treatment, corresponding to a reduction in ESS score, that did not reach statistical significance. Heterogeneity was considerable (τ^2^ = 5.39; χ^2^(5) = 53.07; P < 0.001; I^2^ = 91%), indicating that individual study estimates diverged substantially, a finding that limits the interpretability of the pooled estimate. The only study arm showing a directional advantage for control over active treatment was Högl et al. (2002) [32], which yielded a MD of −2.59 (95% CI −3.91 to −1.27); this negative estimate is consistent with the crossover design and potential carryover effects within that small trial (n = 15 per arm). All other arms favoured active treatment, with MDs ranging from 0.70 [33] to 6.63 [20]. Given that heterogeneity in this domain exceeds the 90% threshold beyond which pooled estimates are considered essentially uninterpretable, the pooled result below is presented as exploratory only, and narrative synthesis is emphasised over the meta-analytic point estimate in the interpretation that follows.

Leave-one-out sensitivity analysis confirmed that the non-significant pooled effect was robust to the removal of any individual study, with estimates ranging from MD 0.88 to 1.99 (all 95% CIs crossing zero) (Fig. S1, Electronic Supplementary Material). The sole exception was omission of Högl et al. (2002), which yielded a MD of 2.51 (95% CI 1.02 to 3.99), the only iteration producing a statistically significant result, indicating a disproportionate influence of this crossover trial on the aggregate estimate (full results in Table 4).

#### Subgroup analysis by pharmacological class

**Fig. 4:**
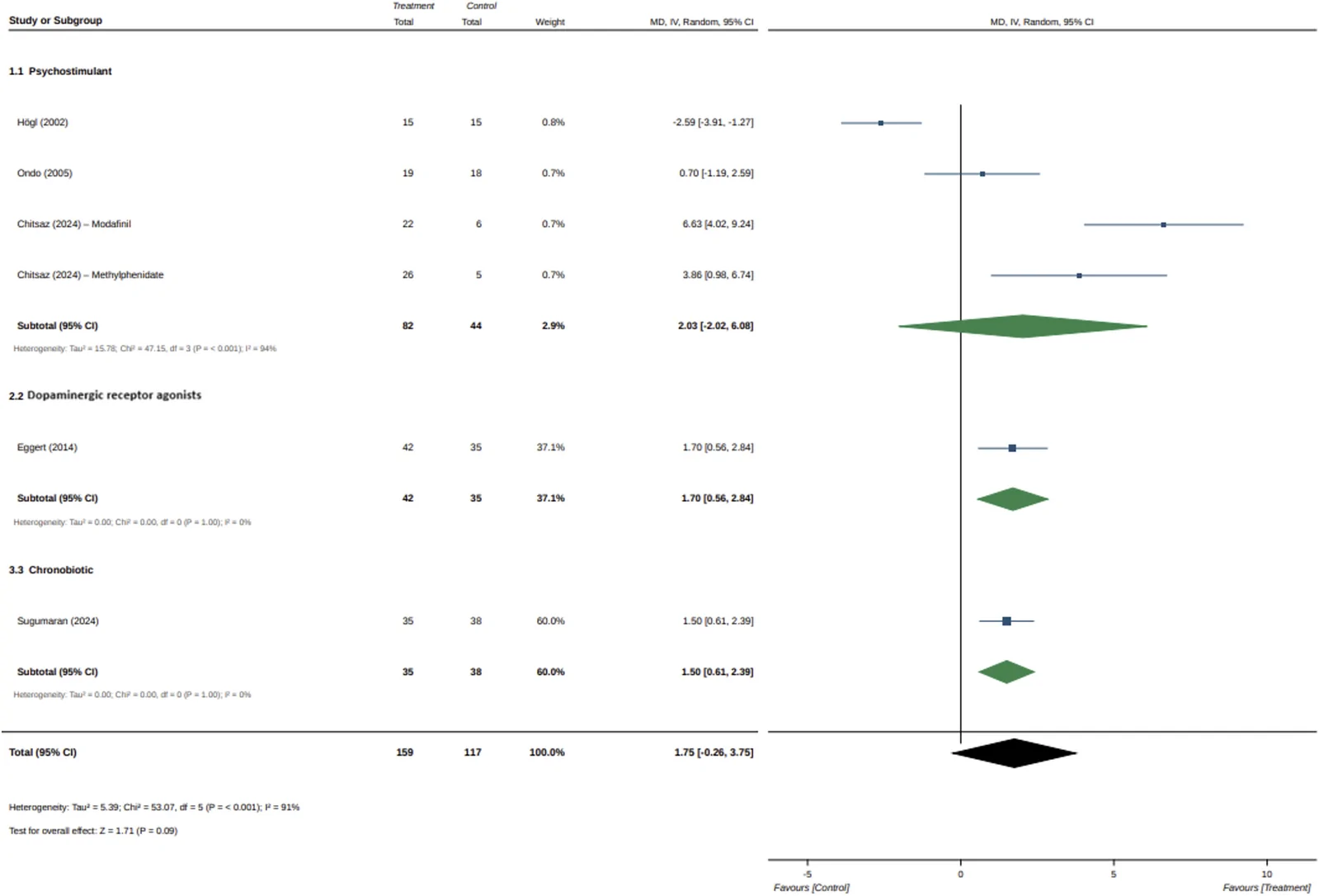
Subgroup analysis for EDS/ESS by pharmacological class (psychostimulants, dopaminergic receptor agonists, chronobiotics)

A pre-specified subgroup analysis stratified trials by pharmacological class: psychostimulants, dopaminergic receptor agonists, and chronobiotics (Fig. 4). Within the psychostimulant subgroup (k = 4), the pooled MD was 2.03 (95% CI −2.02 to 6.08), with very high residual heterogeneity (I^2^ = 94%), driven principally by the discordance between Högl et al. (2002) [32] and the Chitsaz et al. (2024) [20] arms. The dopamine agonist subgroup comprised a single trial [31], yielding a MD of 1.70 (95% CI 0.56 to 2.84), the only pharmacological class with a nominally statistically significant estimate and homogeneous within-group variance (I^2^ = 0%). The chronobiotic subgroup also comprised a single trial [47], with MD = 1.50 (95% CI 0.61 to 2.39). As the dopamine agonist and chronobiotic subgroups each rest on a single trial, these nominally significant estimates should be regarded as hypothesis-generating rather than confirmatory, and they do not upgrade the overall very low certainty of evidence assigned to the ESS outcome as a whole (Table 3).

#### Subgroup analysis by follow-up duration

**Fig. 5:**
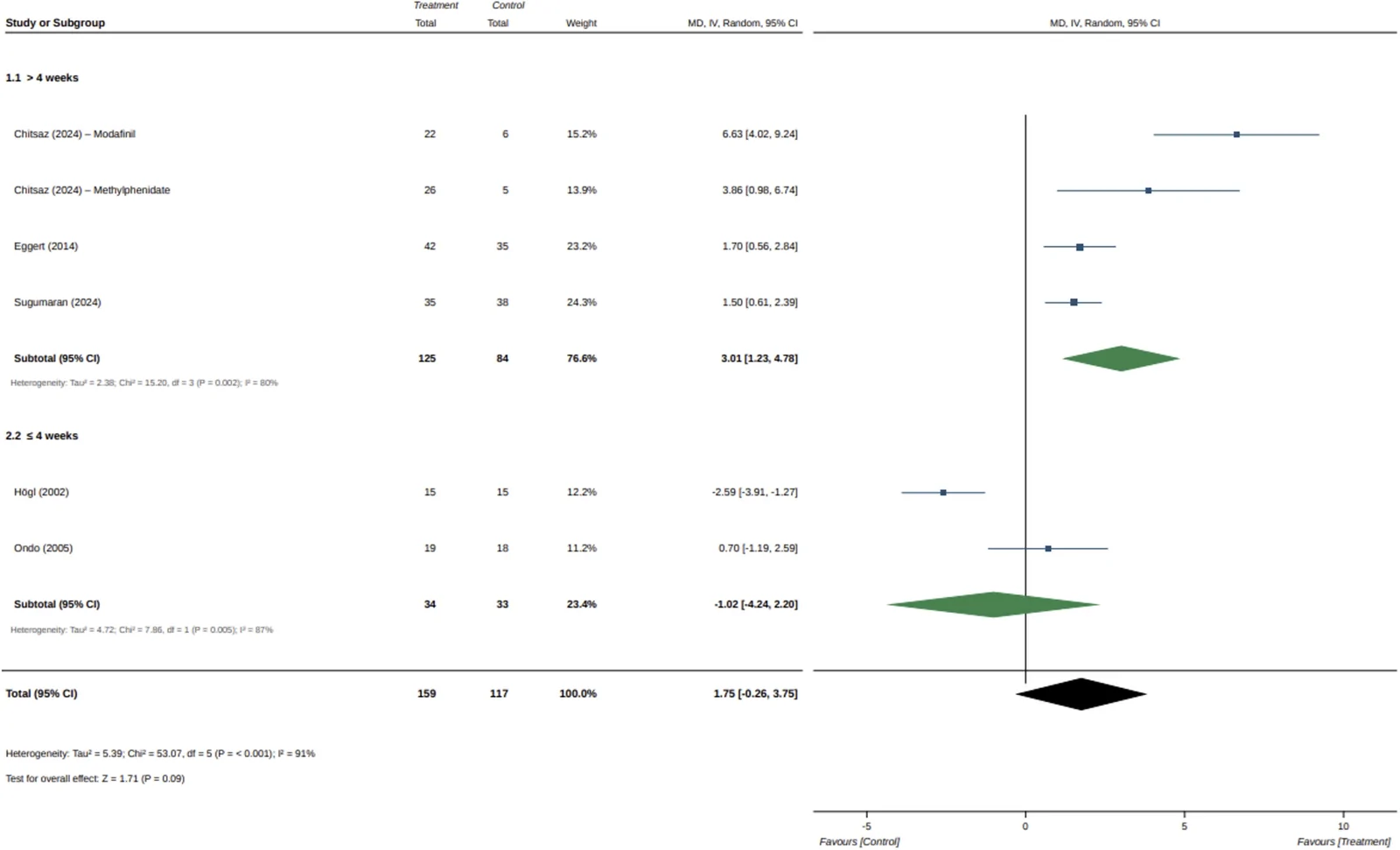
Subgroup analysis for EDS/ESS by follow-up duration

A second pre-specified subgroup analysis examined whether treatment duration moderates the effect on EDS (Fig. 5). Trials with follow-up exceeding four weeks (k = 4) yielded a pooled MD of 3.01 (95% CI 1.23 to 4.78; I^2^ = 80%), which was statistically significant and directionally consistent across all contributing estimates. By contrast, trials with follow-up of four weeks or shorter (k = 2) produced a pooled MD of −1.02 (95% CI −4.24 to 2.20; I^2^ = 87%), with confidence intervals spanning the null (Table 4). A formal test for subgroup differences indicated a statistically significant difference between strata (χ^2^ = 4.59, df = 1, p = 0.032). However, given the small number of trials per subgroup (k = 2 and k = 4) and the substantial residual heterogeneity within each stratum (I^2^ = 87% and 80%, respectively), this result should be interpreted with caution.

#### Overall sleep quality (PSQI)

**Fig. 6:**
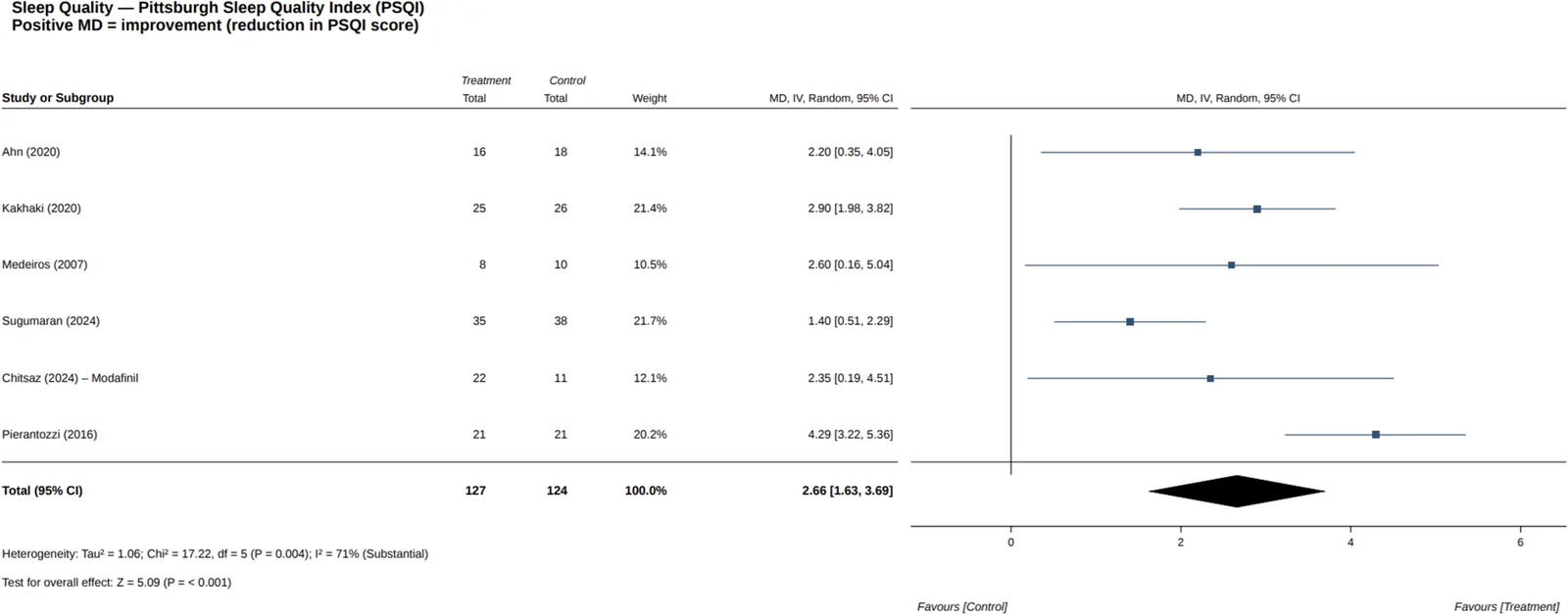
Forest plot for overall sleep quality/PSQI. *positive MD* improvement [reduction in PSQI score]; random-effects model

Six RCTs contributed to the meta-analysis of overall sleep quality assessed by the PSQI, in which higher scores reflect poorer quality sleep and positive MDs indicate improvement (reduction in PSQI score; Fig. 6). The trials evaluated melatonin [26, 38, 44, 47], modafinil [20], and rotigotine transdermal patch [45]. The pooled MD was 2.66 (95% CI 1.63 to 3.69; P < 0.001; Z = 5.09), demonstrating a statistically significant improvement in overall sleep quality under pharmacological treatment compared with placebo. Heterogeneity was substantial (τ^2^ = 1.06; χ^2^(5) = 17.22; P = 0.004; I^2^ = 71%), indicating moderate variation in effect magnitude across agents and populations, while the direction of benefit was consistent across all six contributing trials, with individual MDs ranging from 1.40 [47] to 4.29 [45].

Leave-one-out sensitivity analysis demonstrated that the PSQI meta-analysis was highly robust (Fig. S2, Electronic Supplementary Material). Removal of any individual trial produced pooled estimates ranging from MD 2.20 (95% CI 1.48 to 2.92) to MD 3.11 (95% CI 2.27 to 3.95), with all 95% confidence intervals entirely excluding the null.

#### Subgroup analysis by pharmacological class

**Fig. 7:**
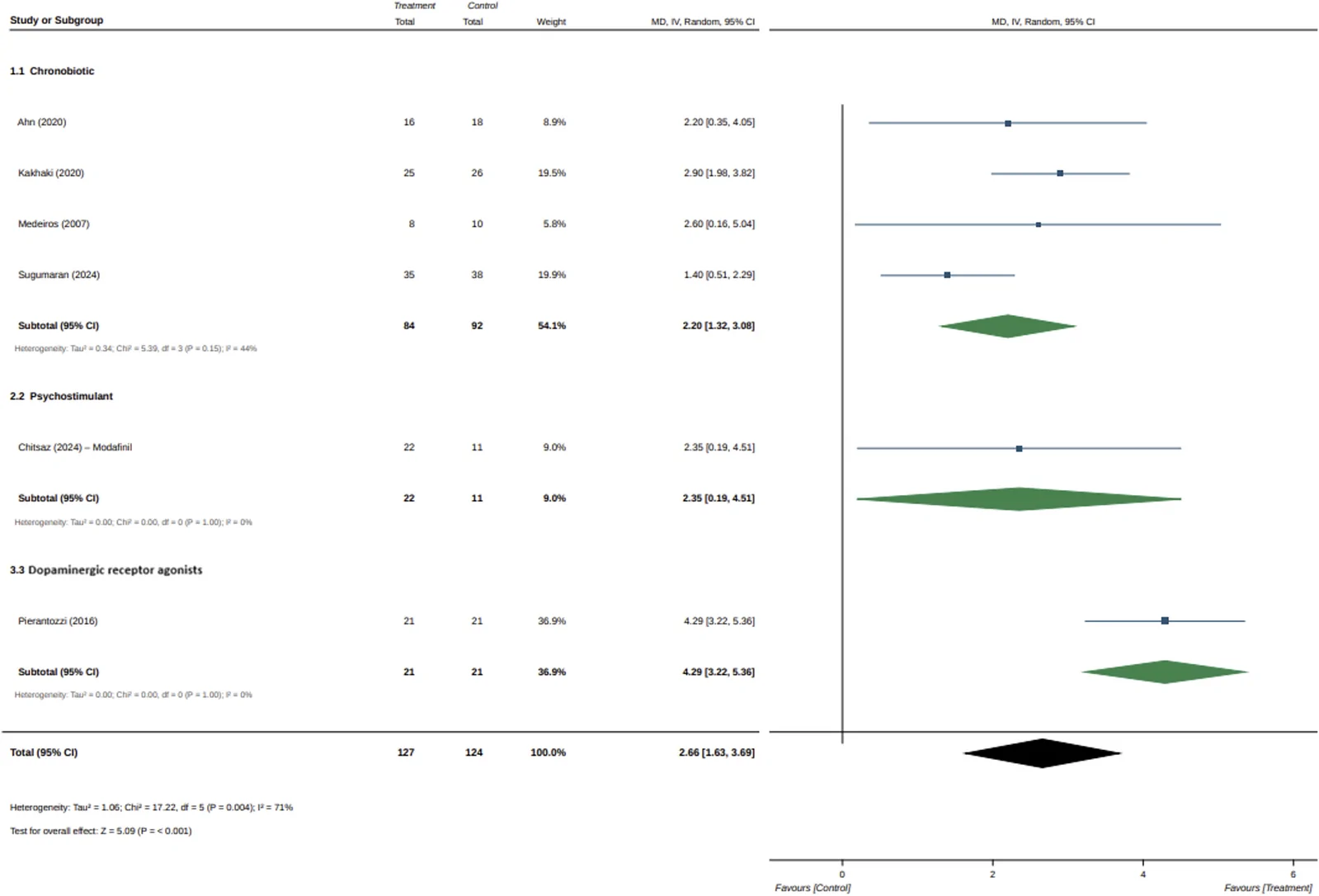
Subgroup analysis for sleep quality/PSQI by pharmacological class

Subgroup analysis stratified by pharmacological class revealed consistent benefit across all three categories assessed (Fig. 7). Chronobiotics (k = 4) produced a pooled MD of 2.20 (95% CI 1.32 to 3.08; I^2^ = 44%), with moderate and statistically non-significant residual heterogeneity (χ^2^(3) = 5.39; P = 0.15), suggesting that melatonin preparations as a class exert a consistent moderate benefit on sleep quality in PD.

#### Insomnia symptoms (PDSS)

**Fig. 8:**
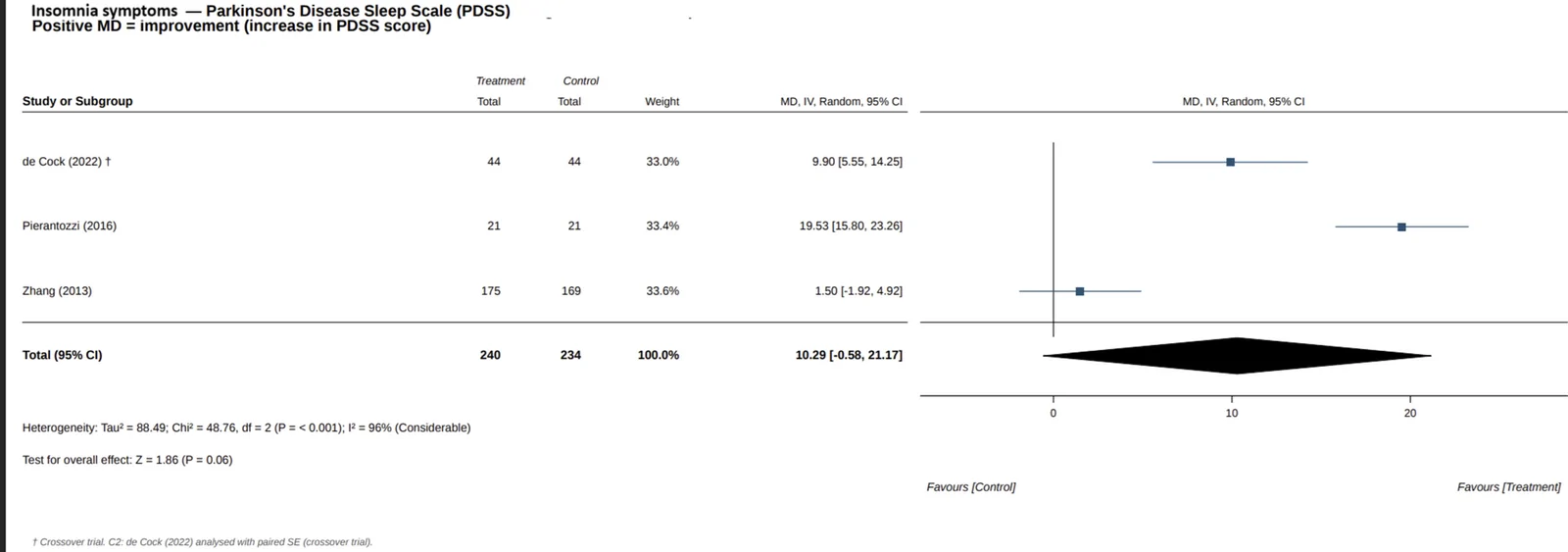
Forest plot for insomnia/PDSS. *positive MD* improvement [increase in PDSS score]; random-effects model. ^†^Crossover trial. C2: De Cock (2022) analysed with paired SE (crossover trial)

Three RCTs contributed to the meta-analysis of insomnia symptom severity assessed by the PDSS, in which higher scores reflect better nocturnal sleep and positive MDs indicate improvement (Fig. 8). Heterogeneity was considerable and statistically significant (τ^2^ = 88.49; χ^2^(2) = 48.76; P < 0.001; I^2^ = 96%), driven by substantial divergence between individual study estimates: MD = 9.90 (95% CI 5.55 to 14.25) [43]; MD = 19.53 (95% CI 15.80 to 23.26) [45]; MD = 1.50 (95% CI −1.92 to 4.92) [49]. As with the ESS domain above, heterogeneity in this domain far exceeds the 90% threshold; the pooled estimate is therefore presented as exploratory only, with narrative synthesis emphasised in interpretation.

Leave-one-out sensitivity analysis indicated that the insomnia symptom/PDSS pooled estimate was sensitive to individual study removal (Fig. S3, Electronic Supplementary Material; full results in Table 4).

#### RBD (PDSS-2)

**Fig. 9:**
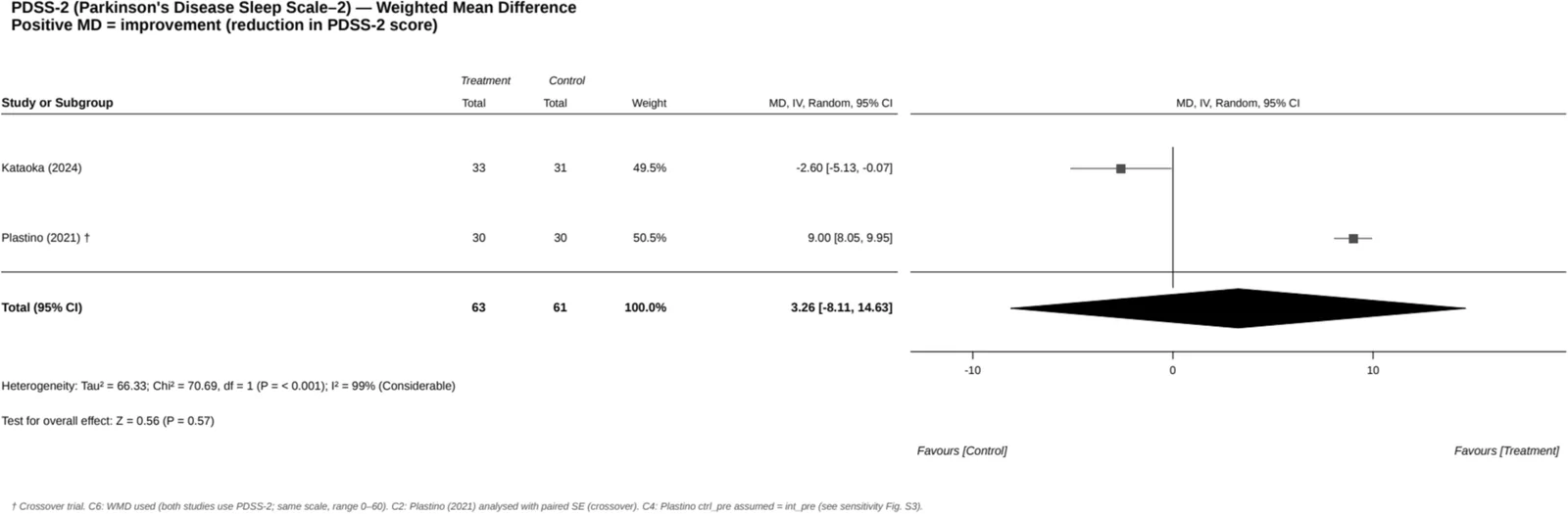
Forest plot for REM sleep behaviour disorder/PDSS-2. *positive MD* improvement [reduction in PDSS-2 score]; random-effects model. ^†^Crossover trial. C6: WMD used (both studies use PDSS-2; same scale, range 0–60). C2: Plastino (2021) analysed with paired SE (crossover)

Two RCTs contributed to the meta-analysis of RBD severity assessed by the PDSS-2 (Fig. 9). The included trials evaluated zonisamide [39] and safinamide [41]. Both studies used the PDSS-2 as a primary or secondary RBD-related outcome, enabling a weighted mean difference to be computed on the common metric. The pooled MD was 3.26 (95% CI −8.11 to 14.63; P = 0.57; Z = 0.56), which did not reach statistical significance, reflecting near-complete cancellation of the two divergent individual estimates. It should also be noted that RBD diagnosis in both contributing trials was not established using International Classification of Sleep Disorders (ICSD) criteria, and severity was inferred from the PDSS-2, rather than a dedicated, validated RBD severity instrument; this indirectness further limits the interpretability of the pooled estimate and is noted as a limitation below. Given heterogeneity of this magnitude, this pooled estimate is presented as exploratory only and should not be interpreted as evidence for or against a treatment effect; narrative synthesis is the appropriate basis for conclusions in this domain.

Leave-one-out analysis was not performed owing to the minimum required number of studies (k = 2). Additional RCTs addressing RBD with compatible outcome instruments are required before a meaningful synthesis is feasible.

#### Safety and tolerability

**Fig. 10:**
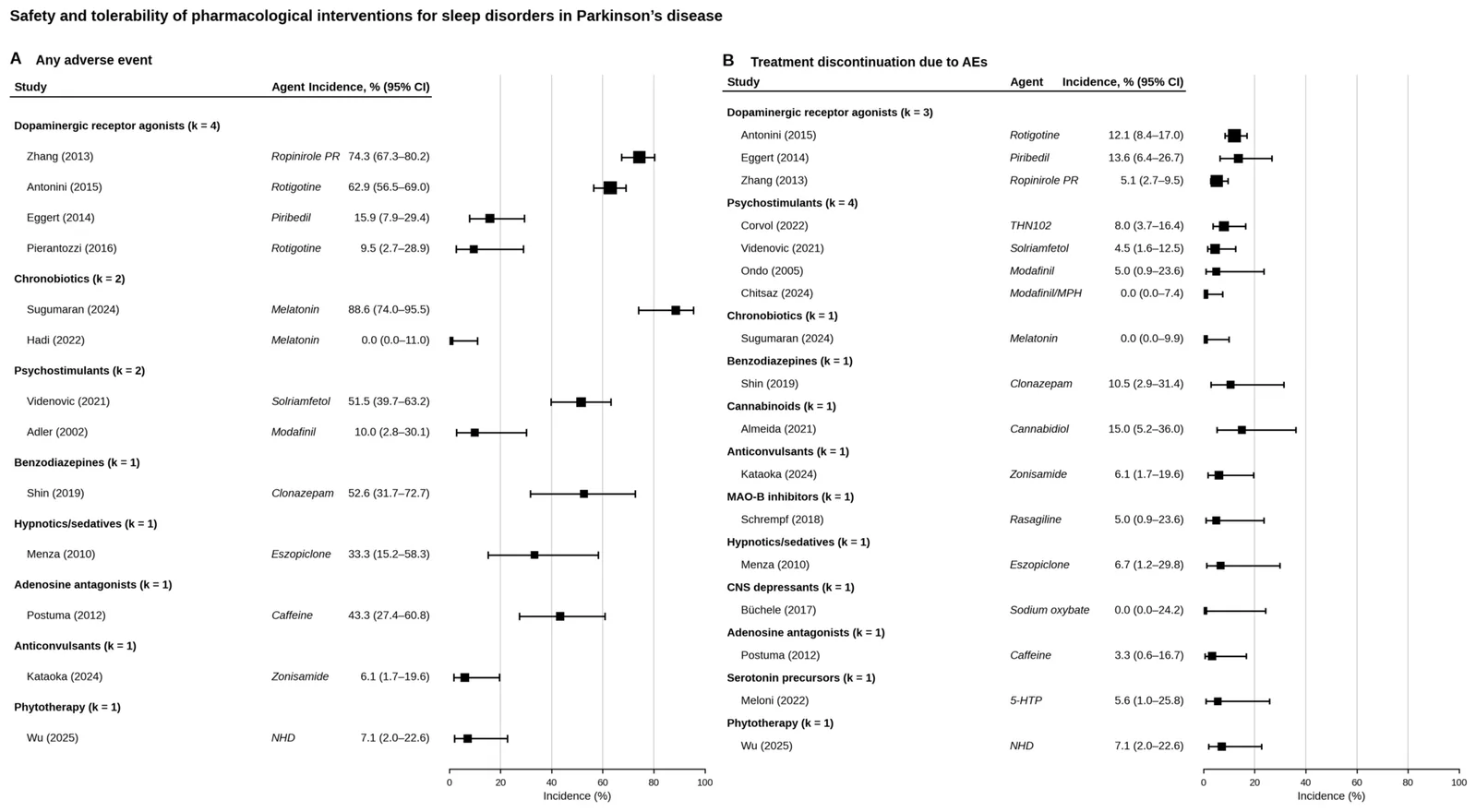
Safety and tolerability of pharmacological interventions for sleep disorders in PD. Panel A: any adverse event incidence (%). Panel B: treatment discontinuation due to adverse events (%). 95% CIs computed by Wilson score method

The incidence of any adverse event (AE) and treatment discontinuation due to AEs were characterised narratively and graphically across all included studies, stratified by pharmacological class (Fig. 10). Formal quantitative pooling of safety data was not performed owing to the heterogeneity of AE definitions, reporting granularity, and monitoring periods across trials; incidence proportions with 95% Wilson score confidence intervals are displayed for each study with extractable binary data.

The highest rates of any AE were observed with dopaminergic receptor agonists: ropinirole prolonged-release (74.3%; 95%CI 67.3–80.2) [49], rotigotine (62.9%; 95%CI56.5–69.0) [23], and ropinirole/rotigotine (9.5%; 95%CI 2.7–28.9) [45], the latter reflecting a shorter exposure period (10 days). The psychostimulant class showed marked heterogeneity: solriamfetol (51.6%; 95%CI 39.6–63.4) [35] and modafinil (10.0%; 95%CI 2.8–30.1) [28]. Amongst chronobiotics, melatonin incidence varied substantially between trials: 88.6% (74.0–95.5%) in Sugumaran et al. (2024) [47] versus 0.0% (0.0–11.0%) in Hadi et al. (2022) [21], reflecting differences in AE ascertainment methodology and the spectrum of events captured. Clonazepam [42] and eszopiclone [24] showed rates of 52.6% (31.7–72.7%) and 33.3% (15.2–58.3%), respectively. Agents with notably low AE rates, consistent with favourable short-term tolerability, included zonisamide (6.1%; 95%CI 1.7–19.6) [39], nyctinastic herbs decoction (7.1%; 95%CI 2.0–22.6) [48], and piribedil (15.9%; 95%CI 7.9–29.4) [31].

Rates of treatment discontinuation attributable to AEs were generally low across most pharmacological classes. Amongst dopaminergic receptor agonists, rotigotine discontinuation rates were 12.1% (8.4–17.0%) [23] and 13.6% (6.4–26.7%) for piribedil [31], while ropinirole yielded 5.1% (2.7–9.5%) [49]. Psychostimulants showed a range from 0.0% (0.0–7.4%) [20] to 8.0% (3.7–16.4%) [30], with modafinil at 5.0% (0.9–23.6%) [33]. Cannabidiol showed 15.0% (5.2–36.0%) [36], the highest discontinuation rate across all classes with more than one contributing study. Clonazepam [42] discontinuation due to AEs was 10.5% (2.9–31.4%). Sodium oxybate [29], adenosine antagonists/caffeine [34], and nyctinastic herbs decoction [48] all demonstrated low or zero discontinuation rates, indicating acceptable tolerability within the duration of these trials. Across the full dataset, no unexpected or severe safety signals were identified that had not previously been characterised in the broader pharmacological literature for each agent class in PD, within the limited scope and duration of the included trials (29 trials, 1,913 participants, treatment durations of 2–24 weeks); this reassurance should not be extrapolated to longer-term or larger-scale use.

#### Assessment of publication bias

Publication bias was assessed graphically for the EDS/ESS and PSQI meta-analyses (k = 6 each) (Figs. S4 and S5, Electronic Supplementary Material). Given the limited number of studies, funnel plots were inspected descriptively for the two largest outcome domains (k=6 each). No formal test for funnel-plot asymmetry was performed as all meta-analyses contained fewer than 10 studies. Visual findings were considered exploratory and were not interpreted as evidence for or against publication bias. The EDS/ESS funnel plot showed some asymmetry, with one arm falling outside the 95% pseudo-confidence interval, suggesting possible small-study effects. The PSQI funnel plot was more symmetric, providing tentative reassurance against major publication bias. Assessment was not feasible for the insomnia symptoms/PDSS and RBD/PDSS-2 analyses given the limited number of contributing studies.

## Discussion

This systematic review and meta-analysis of 29 randomised controlled trials enrolling 1913 participants provides a comprehensive synthesis of pharmacological interventions for sleep disorders in PD, stratified by sleep phenotype. The central finding is that treatment effects are phenotype-dependent: statistically significant and directionally consistent improvement was observed for overall sleep quality, whereas evidence for EDS, insomnia symptoms, and RBD was either non-significant, statistically fragile, or rendered uninterpretable by high between-study heterogeneity, and for these latter three domains (I^2^ ≥ 91%) pooled estimates are presented as exploratory only, with narrative synthesis emphasised over meta-analytic pooling. Across all outcome domains, the certainty of evidence ranged from low to very low, reflecting the methodological limitations pervasive in the available trial base, and the finding that fewer than one in five trials was judged at low risk of bias substantially limits confidence in all pooled estimates discussed below, irrespective of statistical significance.

For EDS, the pooled ESS estimate favoured pharmacological treatment (MD 1.75; 95% CI −0.26 to 3.75) but did not reach statistical significance (P = 0.09), with considerable heterogeneity (I^2^ = 91%; GRADE: very low certainty). This is consistent with prior meta-analytic work [15, 16], which similarly concluded that the benefit of wake-promoting agents on subjective EDS in PD lacks robust pooled support despite directionally favourable individual estimates. The disproportionate influence of Högl et al. (2002) [32] on the aggregate result is noteworthy: this small crossover trial was the only arm producing a negative MD, and its omission in leave-one-out analysis was the only iteration yielding a statistically significant pooled estimate (MD 2.51; 95% CI 1.02 to 3.99). The anomalous direction of its estimate is consistent with carry-over effects inherent to crossover designs with inadequate washout, a concern supported by the RoB 2 assessment. The overall non-significant pooled effect should therefore be interpreted in light of this influential outlier rather than taken as evidence of treatment futility. Subgroup analysis by pharmacological class identified the dopaminergic receptor agonist piribedil [31] and the chronobiotic melatonin [47] as the only agents with nominally significant individual estimates and low within-class heterogeneity. Within the psychostimulant class, residual heterogeneity remained very high (I^2^ = 94%), driven mainly by discordance between the Högl et al. (2002) [32] and Chitsaz et al. (2024) [20] arms. As the piribedil and melatonin subgroup estimates each rest on a single trial, they should be interpreted as hypothesis-generating and do not upgrade the overall very low GRADE certainty assigned to the ESS outcome (Table 3). Subgroup analysis by treatment duration suggested that pharmacological benefit on EDS may require more than four weeks to emerge (MD 3.01; 95% CI 1.23 to 4.78 for trials exceeding four weeks versus MD −1.02; 95% CI −4.24 to 2.20 for shorter trials). However, this finding rests on few independent RCTs per stratum (three RCTs/four estimates versus two RCTs/two estimates), unstable heterogeneity estimates, and a post hoc dichotomization of duration; it should be regarded as hypothesis-generating rather than as evidence that treatment duration causally modifies efficacy.

For overall sleep quality, the pooled PSQI analysis demonstrated statistically significant improvement under pharmacological treatment (MD 2.66; 95% CI 1.63 to 3.69; P < 0.001; I^2^ = 71%), with directional consistency across all six contributing trials (GRADE: low certainty). This magnitude approximates the minimal clinically important difference reported in comparable populations [8] and is unlikely to reflect random variation alone. The finding aligns with recent meta-analytic evidence focused on melatonin [17], though the present analysis encompasses a broader pharmacological spectrum. Sensitivity analysis confirmed that this result was highly robust, with leave-one-out estimates ranging from MD 2.20 to 3.11, all with 95% confidence intervals entirely excluding the null (Fig. S2, Electronic Supplementary Material). Subgroup analysis indicated consistent benefit across chronobiotics (MD 2.20; 95% CI 1.32 to 3.08; I^2^ = 44%), with the largest individual effect observed for rotigotine transdermal patch (MD 4.29; 95% CI 3.22 to 5.36) [45], a finding biologically plausible given the capacity of dopaminergic receptor agonists to improve nocturnal sleep architecture via D3 receptor activity in the basal ganglia and hypothalamus [11, 12]. However, as rotigotine is primarily prescribed to treat the motor symptoms of PD rather than sleep disturbance specifically, we cannot exclude the possibility that part of the observed PSQI improvement reflects secondary benefits of better nocturnal motor control (e.g., reduced nocturnal akinesia, rigidity, and repositioning difficulty) rather than a direct sleep-specific pharmacological effect; this potential confound should be borne in mind when attributing the magnitude of sleep-quality benefit to dopaminergic agents, and is noted as a limitation below.

For insomnia symptoms assessed with the PDSS, the pooled estimate was directionally favourable (MD 10.29; 95% CI −0.58 to 21.17; P = 0.06) but narrowly non-significant, with very high heterogeneity (I^2^ = 96%) across three trials evaluating pharmacologically distinct agents in substantially different populations (GRADE: very low certainty). Leave-one-out sensitivity analysis confirmed that the aggregate result was highly sensitive to individual study removal, with statistical significance achieved only upon omission of Pierantozzi et al. (2016) [45], driven in that iteration by the weight of Zhang et al. (2013) [49] (Fig. S3, Electronic Supplementary Material). The high heterogeneity is mechanistically interpretable given the divergent targets, disease stages, and treatment durations across contributing trials. Under these conditions, a reliable, pooled estimate cannot be derived, and the apparent trend toward improvement must be interpreted with considerable caution.

For RBD assessed with the PDSS-2, the meta-analysis was limited to two trials and yielded a non-significant pooled estimate (MD 3.26; 95% CI −8.11 to 14.63; P = 0.57) with very high heterogeneity (I^2^ = 99%; GRADE: very low certainty). The two contributing studies produced directionally opposite estimates: zonisamide [39] was associated with a statistically significant worsening of PDSS-2 scores, interpreted by the original authors as potentially reflecting drug-induced REM sleep suppression paradoxically increasing motor phenomena captured by the scale, whereas safinamide [41] produced a large, statistically significant improvement. The pooled estimate is therefore clinically uninformative and should be regarded as descriptive of the heterogeneous evidence base rather than as evidence for or against any treatment effect. This uninformativeness is compounded by the fact that RBD in both contributing trials was not diagnosed according to ICSD criteria and that severity was inferred from the PDSS-2, a global Parkinson's sleep scale rather than an RBD-specific validated instrument. The remaining RBD trials could not contribute to formal pooled analysis owing to the use of incompatible outcome instruments [36, 37, 40, 42], reflecting the absence of a universally adopted primary outcome measure for pharmacological RCTs in RBD, a recognised gap in the field [6, 14].

Across all pharmacological classes, no unexpected or severe safety signals were identified beyond those previously characterised in the broader literature, although this reassurance is bounded by the limited scope and duration of the included trials (29 trials, 1913 participants, 2–24 weeks of exposure) and should not be extrapolated to longer-term or wider clinical use. Dopaminergic receptor agonists were associated with the highest rates of any AE, consistent with known class effects including nausea, orthostatic hypotension, and impulse control disorders. Treatment discontinuation rates due to adverse events were generally low, with cannabidiol showing the highest discontinuation rate amongst agents with more than one contributing study (15.0%) [36]. The heterogeneity in AE ascertainment methodology across trials limits formal quantitative synthesis and should be addressed in future RCTs through standardised safety reporting frameworks.

### Clinical implications

Notwithstanding the low-to-very-low overall certainty of evidence, some provisional practical observations can be drawn for clinicians. For overall sleep quality, melatonin (3–10 mg/day) and prolonged-release melatonin (2 mg/day) showed the most consistent benefit, with only moderate, non-significant residual heterogeneity within the chronobiotic subgroup (I^2^ = 44%); the rotigotine transdermal patch (16 mg/24 h) produced the single largest effect, albeit with the motor-symptom confound noted above. For EDS, no drug can be recommended with confidence: only piribedil (100–300 mg/day) and melatonin showed nominally significant benefit, each from a single trial, whereas psychostimulants (modafinil 100–400 mg/day, methylphenidate 10 mg/day) produced inconsistent results, and any signal of benefit appeared more likely with treatment durations exceeding four weeks, though this rests on only three RCTs versus two and should not guide dosing-duration decisions in practice. For insomnia symptoms and RBD, no specific agent or dose can currently be recommended, given the very low certainty and instability of the pooled estimates in both domains. These observations should be regarded as hypothesis-generating and provisional, pending adequately powered, phenotype-specific trials.

This review has limitations that must be considered when interpreting the findings. Only 17.2% of included trials were judged to have low overall risk of bias, with the majority raising some concerns or classified as high risk, primarily due to limitations in randomisation reporting and handling of missing outcome data; this substantially limits confidence in the pooled findings reported above. Clinical heterogeneity across trials, including the grouping within pharmacological pooling of mechanistically distinct drug classes (e.g., psychostimulants, dopaminergic agonists, chronobiotics, and MAO-B inhibitors), variable treatment durations (2–24 weeks), and differing outcome instruments and PD disease stages/severities, encompassing differences in disease stage, concomitant antiparkinsonian therapy, sleep phenotype definition, and treatment duration, limits generalisability and likely contributes to the statistical heterogeneity observed; for this reason, narrative synthesis rather than the pooled point estimate should guide interpretation for the three domains with I^2^ > 90% (ESS, insomnia symptoms, RBD). The small number of contributing studies for several outcomes renders the pooled estimates sensitive to individual trials, as demonstrated by the leave-one-out analyses (Table 4). The RBD/PDSS-2 pooled estimate is further limited by the absence of ICSD-based diagnosis and by reliance on a non-RBD-specific severity scale in both contributing trials, and the largest single-trial effect on sleep quality (rotigotine) may be partly confounded by concurrent improvement in PD motor symptoms rather than a purely sleep-specific drug effect, as discussed above. Heterogeneous adverse-event definitions and reporting granularity across trials (e.g., melatonin-related adverse event rates ranging from 0% to 88.6% across studies) precluded quantitative pooling of safety data and limit the strength of the safety conclusions reached.

Finally, publication bias cannot be formally excluded for any of the four domains given the insufficient number of studies (k < 10) for Egger’s regression testing in all four domains; funnel plot inspection for the EDS/ESS and PSQI analyses suggested possible small-study effects for EDS but tentative reassurance for sleep quality, and this uncertainty should be regarded as an open limitation rather than a ruled-out concern. Strengths of this review include its broad phenotypic scope, rigorous adherence to Cochrane methodology, pre-specified sensitivity and subgroup analyses, transparent statistical handling of crossover and multi-arm trials, independent dual-reviewer screening and risk of bias assessment, and systematic application of the GRADE framework to contextualise the certainty of evidence for each primary outcome.

Future RCTs in this field should prioritise prospective phenotypic stratification of sleep disorders, adoption of harmonised primary outcome instruments across studies, and adequate sample sizes to detect clinically meaningful effects. Head-to-head comparisons between pharmacological classes, exploration of combination strategies, and inclusion of polysomnographic endpoints alongside patient-reported outcomes remain largely unaddressed in the current evidence base and represent priority directions for the field.

## Conclusions

This systematic review and meta-analysis of 29 RCTs provides phenotype-stratified evidence on the pharmacological management of sleep disorders in PD. Pharmacological treatment may produce improvement (low-certainty evidence) in overall sleep quality, with consistent benefit observed across chronobiotic agents and dopaminergic receptor agonists, noting that the largest single-trial effect (rotigotine) may be partly confounded by concurrent motor symptom improvement. Evidence for excessive daytime sleepiness is directionally favourable but statistically non-robust at the pooled level; in exploratory subgroup analysis, longer treatment durations appeared more likely to yield meaningful benefit, although this observation is based on few RCTs (three versus two, contributing four versus two effect estimates) and should be regarded as hypothesis-generating rather than a clinical recommendation. For insomnia symptoms and RBD, the available evidence is insufficient to support definitive conclusions, owing to high between-study heterogeneity, a limited number of eligible trials, and the absence of harmonised outcome instruments across studies. The overall quality of the evidence base is limited, with fewer than one in five trials judged at low risk of bias. These findings underscore the need for adequately powered, phenotype-specific randomised trials with standardised outcome measures, pre-specified safety reporting, and sufficient treatment duration to enable reliable and clinically actionable conclusions.

## Supplementary Information

Below is the link to the electronic supplementary material.

Supplementary file1 (PNG 104 KB)

Supplementary file2 (PNG 99 KB)

Supplementary file3 (PNG 81 KB)

Supplementary file4 (PNG 82 KB)

Supplementary file5 (PNG 73 KB)

## Data Availability

All data generated or analysed during this study are included in this submitted article. The datasets used and/or analysed during the current study are available from the corresponding author on reasonable request.

## Code availability

The custom python code for meta-analysis is available on request from the corresponding author.
